# Impact of Acute and Chronic Adverse Events on Quality of Life in Cutaneous Melanoma

**DOI:** 10.3390/cancers18162619

**Published:** 2026-08-14

**Authors:** Ana-Maria Zamfirescu Deryder, Liviu Bîlteanu, Vlad-Luca Moga, Antonia-Ruxandra Folea, Anca Zamfirescu, Radu-Valeriu Toma, Serban-Andrei Marinescu, Steluta Barascu, Andreea-Iren Șerban, Rodica Anghel

**Affiliations:** 1 Faculty of General Medicine, Carol Davila University of Medicine and Pharmacy, 8 Eroii Sanitari Street, 050474 Bucharest, Romania; ana-maria.zamfirescu@drd.umfcd.ro (A.-M.Z.D.); vlad-luca.moga@drd.umfcd.ro (V.-L.M.); antonia-ruxandra.folea@drd.umfcd.ro (A.-R.F.); radu.toma@umfcd.ro (R.-V.T.); serban.marinescu@yahoo.com (S.-A.M.); rodica.anghel@umfcd.ro (R.A.); 2 Oncological Institute “Alexandru Trestioreanu” Bucharest, 252 Soseaua Fundeni, 022328 Bucharest, Romania; ancamzamfirescu@gmail.com (A.Z.); stelutahuzuu@yahoo.com (S.B.); 3 Faculty of Biology, University of Bucharest, 91-95 Splaiul Independentei, 050095 Bucharest, Romania; andreea-iren.serban@fmvb.usamv.ro; 4 Laboratory for Molecular Nanotechnologies, National Institute for Research and Development in Microtechnologies—MT Bucharest, 126A, Erou Iancu Nicolae Street, 077190 Voluntari, Romania; 5 Department of Preclinical Sciences, Faculty of Veterinary Medicine, University of Agronomic Sciences and Veterinary Medicine, 105 Splaiul Independentei, 050097 Bucharest, Romania

**Keywords:** cutaneous melanoma, health-related quality of life, immune checkpoint inhibitors, targeted therapies, immune-related adverse events, survivorship, financial toxicity, neoadjuvant therapy

## Abstract

**Background/Objectives:** The advent of immune checkpoint inhibitors (ICIs) and targeted therapies has revolutionized advanced cutaneous melanoma care, shifting it from an acutely fatal illness to a chronic, manageable condition. While extending survival, these modern therapies introduce a new spectrum of persistent immune-related adverse events (irAEs) and long-term toxicities. This study aims to explore the “Toxicity-HRQoL Paradox” to comprehensively evaluate the true price of survival across the melanoma treatment continuum. **Methods:** This narrative review, based on a systematic database search, synthesizes quantitative patient-reported outcomes, health-related quality of life (HRQoL) trajectories, and health-economic data. It evaluates the impact of surgical, regional, and modern systemic therapies utilizing data derived from validated instruments, including the European Organization for Research and Treatment of Cancer Quality of Life Questionnaire Core 30 (EORTC QLQ-C30), EuroQol 5 Dimensions (EQ-5D), Functional Assessment of Cancer Therapy-Melanoma (FACT-M), and comprehensive score for financial toxicity (COST). Results: Historically, extensive locoregional surgeries and high-dose interferons caused profound, debilitating drops in physical and overall QoL. Today, modern systemic therapies are associated with high rates of severe (Grade ≥ 3) acute toxicities and irreversible chronic conditions, such as permanent endocrinopathies. Despite this, global HRQoL frequently remains stable or even improves. This “toxicity-HRQoL paradox” occurs because the profound psychological relief of durable disease control effectively offsets the physical symptom burden. Furthermore, neoadjuvant ICI approaches demonstrate superior long-term HRQoL by enabling tailored surgical de-escalation. Ultimately, extended survivorship unmasks hidden burdens, revealing high prevalences of fear of cancer recurrence (FCR) (81–86%) and substantial financial toxicity. **Conclusions:** Despite the severe physical toxicities of modern melanoma treatments, the psychological benefit of survival largely preserves overall HRQoL. However, optimizing the modern survivorship experience requires the widespread adoption of melanoma-specific HRQoL tools and proactive, multidisciplinary care models to manage chronic toxicities, psychosocial distress, and financial burdens.

## Simple Summary

Recent medical advancements have transformed advanced melanoma from a rapidly fatal illness into a manageable chronic condition. While patients are living significantly longer, they now face new challenges, including lifelong side effects from modern therapies, ongoing anxiety about cancer recurrence, and severe financial strain. This research aims to explore the true “price of survival” by comprehensively evaluating how these lasting physical and emotional burdens impact a patient’s daily well-being. Surprisingly, many patients report a stable quality of life because the profound relief of surviving often outweighs the negative physical side effects. The findings from this review will impact the medical community by highlighting the urgent need for updated, disease-specific questionnaires to accurately measure these modern side effects, while advocating for the creation of dedicated, multidisciplinary care programs to support long-term melanoma survivors.

## 1. Introduction

### 1.1. The Evolving Landscape of Melanoma Treatment

For decades, cutaneous melanoma, particularly in its advanced stages, was considered a devastating diagnosis with a grim prognosis [1,2,3,4,5,6]. Historically, treatment for metastatic or unresectable disease was largely limited to surgical debulking, conventional cytotoxic chemotherapy (such as dacarbazine), and non-specific cytokine immunotherapies like interleukin-2 (IL-2) and high-dose interferon-alpha [4,5,7,8,9,10,11,12,13,14,15,16,17]. These early systemic treatments demonstrated poor curative potential, low objective response rates (often around 5%), and were heavily associated with severe, dose-limiting toxicities [1,8,9,10,12,15,17,18]. Prior to the modern therapeutic era, patients diagnosed with advanced stage IV melanoma faced a bleak outlook, with a median overall survival of merely 6 to 10 months and a 5-year overall survival rate of less than 10% [1,2,3,4,5,9,11,12,16,17,19].

Over the past decade, a deepened understanding of the genetic mutational burden and immune evasion mechanisms of melanoma has fundamentally revolutionized the treatment landscape [1,2,3,4,6,19,20]. The year 2011 marked a major turning point with the approval of ipilimumab, a monoclonal antibody targeting the CTLA-4 immune checkpoint, and vemurafenib, a targeted inhibitor of the mutated BRAF kinase [7,13,21]. Since then, the therapeutic arsenal has rapidly expanded to include highly effective immune checkpoint inhibitors (ICIs) targeting programmed cell death protein 1 (PD-1), such as nivolumab and pembrolizumab, alongside novel combinations of targeted BRAF and MEK inhibitors (e.g., dabrafenib and trametinib) [13,20,21,22,23,24,25]. By directly inhibiting mutated oncogenic pathways or overcoming tumor-induced immune suppression, these agents have established a new standard of care for patients with both resectable high-risk and advanced melanoma [1,2,3,6,19,20].

The introduction of these novel targeted therapies and immunotherapies has resulted in unprecedented improvements in clinical outcomes and survival rates. The median overall survival for patients with advanced melanoma has increased up to 10-fold, extending from an average of 6 months historically to over 5 years today [1,10]. Furthermore, 5-year overall survival rates for metastatic melanoma have surged from below 10% to an impressive 40% to 50%, with some combination immunotherapy approaches (such as dual blockade with nivolumab and ipilimumab) pushing 5-year survival rates beyond 50% [26]. Currently, approximately 20% of patients treated for metastatic melanoma can survive for up to 10 years following their diagnosis, fundamentally transforming expectations of the disease [19]. Despite these historic gains, therapeutic failure remains a major challenge. Approximately 60% of patients treated with anti-PD-1 monotherapies demonstrate primary (pre-existing) resistance, and 20% to 30% of initial responders eventually develop acquired resistance [26]. This resistance is compounded by the high propensity of advanced melanoma to spread to the central nervous system; brain metastases occur in a substantial subset of patients and are historically associated with a dismal prognosis, necessitating aggressive multidisciplinary interventions such as stereotactic radiosurgery [21,27].

As mortality rates steadily decline and long-term survival becomes a reality for many, advanced melanoma is increasingly shifting from an acutely fatal illness to a chronic, manageable condition. This paradigm shift has brought new clinical challenges to the forefront, particularly regarding the long-term survivorship experience [28,29,30,31,32,33,34]. While modern therapies provide immense clinical and survival benefits, they also expose patients to prolonged periods of treatment and a new spectrum of persistent immune-related adverse events (irAEs) and targeted therapy (TT)-induced toxicities [35,36]. Consequently, evaluating the “price of survival” which includes the profound impact of chronic physical toxicities, psychological distress, and financial burden has become a critical component in the holistic assessment of health-related quality of life (HRQoL) in contemporary melanoma care [22,28,34,37,38,39,40].

### 1.2. The Burden of Treatment: Toxicity and Quality of Life

While the advent of ICIs and targeted therapies (BRAF/MEK inhibitors) has revolutionized melanoma care and dramatically improved survival, these systemic treatments require a delicate balance between achieving therapeutic efficacy and managing treatment-related adverse events (AEs). The unprecedented improvements in progression-free and overall survival often come at the cost of exposing patients to profound physical and psychological toxicities [19,41]. Modern immunotherapies can trigger a unique spectrum of irAEs due to immune activation against healthy host tissues, while targeted therapies are frequently associated with pyrexia, rash, and gastrointestinal symptoms [19,42]. Balancing this efficacy-toxicity trade-off is particularly critical in the adjuvant setting, where patients have already undergone complete macroscopic tumor resection and have a high chance of long-term cure, meaning any treatment-induced harm must be carefully weighed against the statistical reduction in recurrence risk [30,41,43,44,45,46].

Furthermore, while some acute toxicities resolve with treatment breaks or corticosteroids, others such as late-onset endocrinopathies, neuropathies, or chronic skin conditions can be irreversible, introducing lifelong morbidity for survivors [43,47,48,49,50]. Immune checkpoint inhibitors (ICIs) are associated with off-target immune-related adverse events (irAEs) such as colitis, pneumonitis, hepatitis, and permanent endocrinopathies that can emerge even years after treatment discontinuation [47,51]. Conversely, targeted BRAF/MEK inhibitors cause class-specific toxicities, including pyrexia, photosensitivity, cutaneous squamous malignancies, and cardiotoxicity [21,49]. Because these adverse events severely impact patient quality of life, identifying validated predictive biomarkers—including tumor-infiltrating lymphocytes, circulating tumor DNA (ctDNA) kinetics, and circulating tumor cells (CTCs)—is an urgent priority to guide personalized, risk-directed treatment selection and avoid unnecessary toxic exposure [19,48].

Because clear-cut clinical improvements, such as delayed disease progression, do not automatically translate into a noticeable benefit from the patient’s perspective if the adverse-effect profile is high, the routine assessment of HRQoL has become a crucial, standard endpoint in modern oncology trials [23,37,52,53]. HRQoL is a multidimensional construct that encompasses the subjective evaluation of a patient’s physical, emotional, cognitive, and social well-being, effectively summarizing the combined net impact of the disease itself and the interventions used to treat it [23,54,55]. Understanding this metric is essential for patient-centered care, as it helps clinicians and patients mutually determine whether the survival benefits offered by a treatment are offset by an unacceptable deterioration in daily functioning and overall well-being [52,56].

Assessing the patient-reported impact of these therapies requires standardized health-related quality of life (HRQoL) instruments, yet each tool possesses inherent design trade-offs. The European Organization for Research and Treatment of Cancer Quality of Life Questionnaire Core 30 (EORTC QLQ-C30) is the most widely utilized cancer-specific tool, comprising 30 items that generate scores for global health status, five functional scales (physical, role, emotional, cognitive, and social), and multiple symptom scales (including fatigue, pain, and nausea) [37,41,42,53]. While the EORTC QLQ-C30 is robust, it lacks modules specifically designed to capture the unique irAEs of modern immunotherapies, such as specific skin reactions or endocrine dysfunction [41]. To overcome the limitations of generic cancer questionnaires, disease-specific tools like the Functional Assessment of Cancer Therapy-Melanoma (FACT-M) are often used. The FACT-M integrates a general health assessment (FACT-G) with a 24-item melanoma-specific subscale and a surgery scale, providing a more granular look at the specific physical and psychosocial symptoms experienced by melanoma patients [57,58,59].

Generic instruments (e.g., SF-36, EQ-5D) excel at enabling cross-disease comparison and health-economic valuations, but are plagued by ceiling effects and a failure to capture therapy-specific toxicities [29]. Cancer-specific instruments (e.g., EORTC QLQ-C30) offer validated assessments of general functional domains but lack dedicated modules to evaluate modern immunotherapy toxicities (e.g., endocrine dysfunction) or long-term psychosocial sequelae [52]. Melanoma-specific modules (e.g., FACT-M) address localized surgical issues but remain under-equipped to measure the pervasive, chronic psychological uncertainty and fear of recurrence triggered by modern systemic surveillance [22].

Finally, to translate HRQoL into health-economic models, the EuroQol 5 Dimensions (EQ-5D) questionnaire is frequently employed [27,42,60]. The EQ-5D assesses five distinct domains mobility, self-care, usual activities, pain/discomfort, and anxiety/depression and utilizes a Visual Analogue Scale (VAS) to measure self-rated overall health [22,61]. Importantly, responses to the EQ-5D are converted into a preference-based utility score (ranging from 0, representing death, to 1, representing perfect health), which is indispensable for calculating Quality-Adjusted Life Years (QALYs) in cost-effectiveness evaluations [22,60]. Together, the combined use of the EORTC QLQ-C30, FACT-M, and EQ-5D provides a comprehensive, mixed-methods framework to evaluate the true “price of survival” by quantifying both the clinical symptom burden and the economic value of modern melanoma therapies. Table 1 lists the most frequently used measurement tools across the literature, categorizing them into generic, cancer-specific, and melanoma-specific instruments (e.g., EORTC QLQ-C30, FACT-M, EQ-5D, Medical outcomes study short form 36 [SF-36], COST for financial toxicity, and Hospital Anxiety and Depression Scale [HADS]). It summarizes the specific domains each tool covers, highlighting the gap where generic tools sometimes fail to capture melanoma-specific chronic survivorship issues like fear of recurrence.

Cutaneous melanoma arises from the malignant transformation of epidermal melanocytes, a process heavily driven by mutagenic DNA damage induced by solar ultraviolet (UV) radiation [19,68]. At the intracellular level, this transformation is characterized by the hyperactivation of the mitogen-activated protein kinase (MAPK) pathway. Ligand-binding or mutational activation of receptor tyrosine kinases triggers a downstream cascade through RAS, RAF, MEK, and ERK (the MAPK pathway), driving uncontrolled cellular proliferation, survival, and transcription [20,23,68]. Furthermore, the PI3K/AKT/mTOR pathway serves as a secondary, critical survival mechanism, often up-regulated as a compensatory pathway that promotes metabolic adaptation, motility, and resistance to standard therapeutic modalities [19,23]. On a hereditary level, familial melanoma is strongly associated with germline mutations in high-penetrance susceptibility genes, most notably the cyclin-dependent kinase inhibitor 2A (*CDKN2A*) gene, which encodes the p16INK4A and p14ARF cell-cycle regulators [19,23,68]. To facilitate distant metastasis, melanoma cells undergo epithelial-to-mesenchymal-like transitions, actively utilizing embryonic developmental programs to navigate the extracellular matrix, intravasate into lymphatic or hematogenous channels, and colonize distant organs [23].

### 1.3. Identification of the Knowledge Gap and Study Objective

While the therapeutic landscape for cutaneous melanoma has expanded rapidly, the existing literature reviewing patient-reported outcomes tends to remain fragmented. Current systematic reviews and meta-analyses focus heavily on evaluating HRQoL strictly during the active on-treatment phase, analyzing specific emerging regimens like neoadjuvant treatments, or isolating distinct psychological symptoms [28,29,69]. Although these studies provide invaluable insights into the immediate impact of modern therapies, they often fail to capture the holistic patient journey from diagnosis through extended survivorship. A major knowledge gap exists because the current literature on patient-reported outcomes remains highly fragmented. Prior systematic reviews and meta-analyses are largely restricted to evaluating HRQoL strictly during the active, on-trial treatment phase [29]. By limiting their scope to clinical trial durations, earlier reviews fail to capture the long-term, real-world survivorship continuum, leaving clinicians without a clear understanding of persistent physical, psychological, and financial toxicities that linger years after therapy cessation. This study directly addresses this deficit by synthesizing outcomes across both acute and chronic treatment phases.

Avoiding superposition with previous literature, this review uniquely contrasts the acute, early-onset toxicities of melanoma treatments with the long-term, chronic survivorship burdens spanning the entire treatment continuum. Historically, locoregional interventions such as complete lymph node dissections (CLNDs) and isolated limb perfusions were the primary drivers of morbidity, leaving patients with profound and lasting physical impairments such as chronic lymphedema [58,70,71]. Today, however, the burden has shifted toward systemic complications. Extended survivorship exposes patients to novel chronic burdens, including irreversible and permanent endocrinopathies, late-onset irAEs that manifest months or years after treatment cessation, and severe financial toxicity resulting from the high costs of modern cancer care and prolonged absences from the workforce [36,47,48,64].

While modern systemic advancements have shifted the therapeutic paradigm, surgery-related complications remain a highly significant cause of chronic morbidity. Radical surgical procedures, particularly groin dissections (inguinal and ilio-inguinal lymphadenectomies), are still standard for many stage III patients and frequently lead to severe, treatment-limiting, and irreversible complications such as lower limb lymphedema, chronic pain, and permanent functional restriction [70,72].

To conceptualize this shift, the core objective of this study is to introduce and quantitatively explore the “Toxicity-HRQoL Paradox”. Older, less effective systemic therapies, such as high-dose interferon-alpha, were characterized by severe acute toxicities that caused profound, well-documented deteriorations in patients’ global HRQoL and health utility scores [73,74]. Conversely, highly toxic modern treatments including combined ICIs and targeted BRAF/MEK therapies frequently result in stable or even clinically improved global HRQoL scores, despite inducing high rates of Grade ≥ 3 adverse events [41,42,43,52]. This review will quantitatively synthesize how the unprecedented psychological relief of survival and prolonged recurrence-free intervals effectively offsets the physical symptom burden of modern regimens, offering a new perspective on the true price of survival for patients with cutaneous melanoma. Table 2 synthesizes toxicity profiles across the treatment continuum and demonstrates how modern treatments have shifted the burden toward specific persistent irAEs.

## 2. Search Strategy and Selection Criteria

To ensure transparency, rigor, and reproducibility, the literature search, study selection, and reporting processes of this review were conducted systematically, but this is not a systematic review as per the Preferred Reporting Items for Systematic Reviews and Meta-Analyses (PRISMA) guidelines. A comprehensive literature search was performed in March 2026 using Web of Science, PubMED and Scopus databases, covering studies published between January 1993 and February 2026. The search formula employed the following query to filter relevant articles and abstracts; the search was conducted within the title and abstract fields:

PUBMED:

(“Melanoma”[MeSH Terms] OR “cutaneous melanoma”[Title/Abstract] OR “skin melanoma”[Title/Abstract]) AND (“Prognosis”[MeSH Terms] OR “Prognostic Factors”[Title/Abstract] OR “Survival”[Title/Abstract] OR “Breslow”[Title/Abstract] OR “Ulceration”[Title/Abstract] OR “TNM”[Title/Abstract]) AND (“Therapeutics”[MeSH Terms] OR “Surgery”[Title/Abstract] OR “Interferon”[Title/Abstract] OR “Radiotherapy”[Title/Abstract] OR “Immunotherapy”[Title/Abstract] OR “Targeted therapy”[Title/Abstract]) AND (“Quality of Life”[MeSH Terms] OR “Patient Reported Outcome Measures”[MeSH Terms] OR “QoL”[Title/Abstract] OR “FACT-M”[Title/Abstract] OR “EORTC QLQ”[Title/Abstract]) AND (“Cohort Studies”[MeSH Terms] OR “Retrospective Studies”[MeSH Terms] OR “Prospective Studies”[MeSH Terms] OR “Cross-Sectional Studies”[MeSH Terms])

WOS and SCOPUS:

(“cutaneous melanoma” AND (prognosis OR survival OR “Breslow” OR “TNM”) AND (treatment OR surgery OR interferon OR immunotherapy OR radiotherapy) AND (“quality of life” OR QoL OR “patient-reported”))

This search strategy yielded a large number of records; however, only studies relevant to cutaneous melanoma were selected. Eligible studies included those evaluating prognosis, survival outcomes, treatment modalities (surgery, immunotherapy, targeted therapy, radiotherapy, interferon), and their associated adverse events, with a particular focus on acute and chronic toxicities and immune-related adverse events. Studies reporting health-related quality of life (HRQoL) and patient-reported outcomes using validated instruments (e.g., EORTC QLQ-C30, FACT-M, EQ-5D) were prioritized.

Both prospective and retrospective studies, clinical trials, meta-analyses, reviews, and selected case reports were included if they provided quantitative or clinically relevant data on toxicity and HRQoL. Studies lacking data on adverse events or quality of life, noncutaneous melanoma studies, preclinical research, and articles without full-text availability were excluded. Preclinical research (including in vitro and in vivo animal models) was intentionally excluded because the primary clinical objective of this synthesis is to evaluate human patient-reported outcomes (PROs) and subjective quality of life (HRQoL) domains, which cannot be simulated or self-reported in preclinical settings.

**Figure 1:**
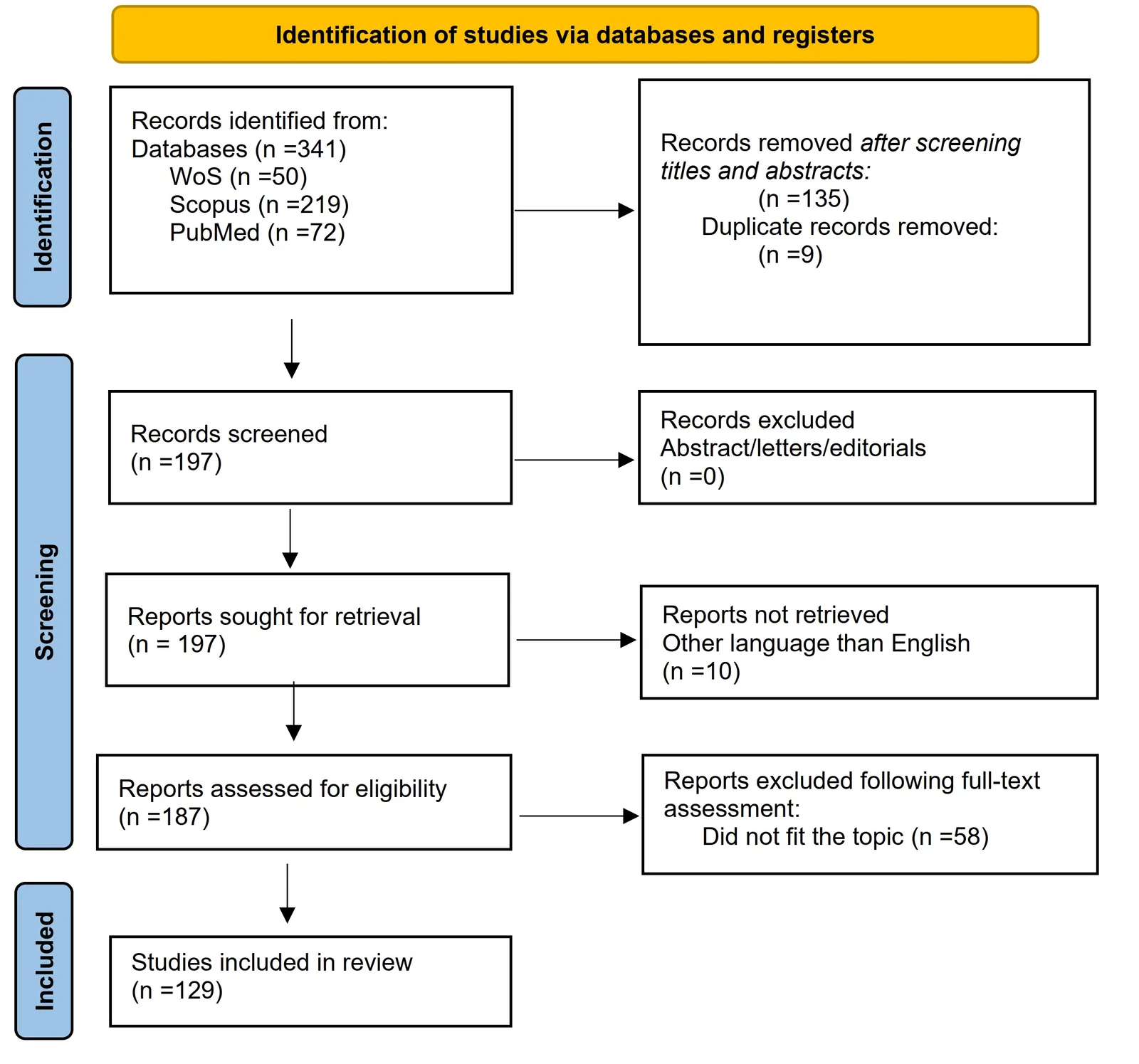
Study flow diagram for systematic search.

The study selection process is summarized in the flowchart presented in Figure 1. To minimize selection bias, the screening of titles, abstracts, and full texts was performed independently and in duplicate by two reviewers (A.M.Z.D. and V.L.M.). Disagreements regarding study eligibility or data extraction were resolved through consensus-seeking discussions, with final arbitration from a senior author (R.A.) when consensus could not be reached. Initially, 341 records were identified across the three databases. After screening titles and abstracts, records that did not meet the scope of this study were excluded, resulting in 206 remaining articles. From these, 9 duplicate records were removed and 10 articles were excluded due to the unavailability of full-text versions in English. Following full-text assessment, a further 58 records were excluded for not aligning with the objective of identifying comprehensive and reliable studies, leaving a total of 129 records for final inclusion. These consisted of 61 original articles, 30 reviews, 6 case reports, 29 clinical trial and 3 guideline.

## 3. Toxicity and HRQoL

### 3.1. Surgical and Regional Therapies

#### 3.1.1. Lymph Node Dissection and Lymphedema

Historically, surgical management of regional lymph nodes was the primary therapeutic intervention for controlling locoregional melanoma, but it carried a substantial risk of long-term physical morbidity. Lymphedema a chronic, debilitating accumulation of lymphatic fluid in the affected extremity is the most significant late surgical complication. The incidence of postoperative lymphedema is heavily dependent on the extent of the surgery and the anatomical site of the nodal basin. Sentinel lymph node biopsy (SLNB) alone is a relatively minimally invasive staging procedure and carries a low risk of lymphedema, generally estimated between 3% and 7% [72,75,86,87]. In stark contrast, CLND or therapeutic lymphadenectomy dramatically increases this risk to 20–30%, with several series reporting even higher rates [72,75,86,87]. The anatomical basin is a critical determinant of morbidity; ilioinguinal dissections are notoriously associated with worse outcomes than axillary dissections. For example, specific institutional data report lymphedema rates of 38% following ilioinguinal lymph node dissection compared to only 12% following axillary dissection [72,86,88]. Furthermore, this complication often worsens progressively over time. Following inguinal lymphadenectomy, lymphedema incidence has been observed to reach 40% at 3 months and escalate to 50% by 12 months post-surgery [72,86,89]. Prospective follow-up studies confirm this disparity, revealing that patient-reported limb edema reaches approximately 55.9% at 6 months post-CLND, compared to 37.5% for those undergoing SLNB alone [71,72].

The chronic morbidity of limb edema exerts a profound and multifaceted impact on long-term physical functioning, cosmesis, and social HRQoL. Patients who develop melanoma-related limb lymphedema consistently report significantly worse global health status, compounded by pronounced deficits in physical, role, and social functioning [70,71,72]. Functionally, limb swelling directly correlates with restricted mobility, chronic regional pain, and difficulties with daily activities such as walking, running, or climbing stairs, which drive sustained decreases in health utility scores [71,90]. Beyond physical limitations, lymphedema severely impacts psychological and social well-being. A distinct interaction with patient demographics has been identified, wherein younger patients and women with lymphedema report disproportionately worse social functioning and significantly higher levels of body image dissatisfaction [70]. These patients also experience higher rates of financial difficulties, potentially due to the need for ongoing decongestive physiotherapy, lifelong compression garments, or a reduced ability to engage in the workforce [70,72,88].

Interestingly, while the localized symptom burden of extensive lymphadenectomy is undeniably high, longitudinal data suggest that a patient’s overall global QoL often stabilizes or rebounds over extended follow-up periods. While patients undergoing axillary CLND report steep initial drops in physical and role functioning [91], longitudinal studies demonstrate that the time required to return to baseline global QoL and physical condition scores can be remarkably similar for patients undergoing CLND compared to those receiving SLNB alone, with recovery typically occurring within 12 to 24 months [92]. Despite this generalized QoL adaptation, patients frequently endure ongoing regional symptoms and limb volume increases that persist for years [72,93]. The recognition of this severe, localized, and irreversible morbidity coupled with pivotal trial data (such as MSLT-II and DeCOG-SLT) demonstrating no overall survival benefit for immediate CLND in patients with sentinel node micrometastases has catalyzed a major paradigm shift in melanoma care. Today, SLNB remains the standard for surgical staging, while morbid CLNDs are increasingly omitted or strictly reserved for highly selected cases with clinically palpable disease that are unresponsive to modern neoadjuvant systemic therapies [75,88,94].

#### 3.1.2. Isolated Limb Perfusion/Infusion (ILP/ILI)

For patients presenting with unresectable, in-transit melanoma metastases of the extremities, ILP and isolated limb infusion (ILI) serve as highly effective locoregional interventions [58,65,94,95]. While these procedures offer substantial palliative and therapeutic benefits yielding overall response rates (ORR) as high as 90%, including complete responses in 58% of cases they are inherently associated with significant acute regional toxicity [58,94]. The delivery of high-dose chemotherapy, most commonly melphalan, directly into the confined vascular circuit of the limb almost universally results in acute grade 1 or 2 toxicities, such as localized erythema, pronounced limb edema, hyperpigmentation, and pain [65]. Furthermore, severe acute regional toxicities (Grade ≥ 3 or Wieberdink grade III–IV, characterized by severe edema and epidermolysis/blistering) are reported in 18% to 36% of patients [65,78]. In rare instances, this intense acute inflammatory reaction can escalate to devastating complications, including acute compartment syndrome, muscle ischemia, renal failure, or the need for limb amputation [76,78].

Beyond the acute postoperative phase, regional therapies, such as ILP and ILI remain highly effective local options for in-transit extremity disease but frequently result in long-term functional disturbances and permanent localized morbidity display a vastly different toxicity profile compared to modern systemic immunotherapies.

The chronic sequelae of these intense regional therapies include muscle inflammation, severe or persistent lymphedema, erythema, compartment syndrome, muscle atrophy, neuropathy, and stinging paresthesias that cause a transient, short-term decline in localized physical functioning through restricted limb mobility [49,58,65,77,78]—they completely avoid systemic complications. In contrast, modern systemic checkpoint inhibitors (ICIs) avoid local limb morbidity but introduce a lifelong risk of chronic, multi-organ immune-related adverse events (irAEs) that can result in persistent, subtle impairments across emotional, social, and general health domains [52,96].

A retrospective evaluation of long-term survivors (with a median follow-up of 14 years post-ILP) demonstrated that approximately half of the patients (49% to 55%) continued to report functional complaints in the treated limb [77]. Additionally, 31% to 38% experienced long-term cosmetic dissatisfaction due to enduring limb asymmetry, skin color changes, or scarring [77]. Alongside these physical disturbances, survivors maintained a high psychological burden, with 77% reporting chronic fear of local disease recurrence and 63% fearing distant metastases [77].

Despite this formidable profile of acute toxicity and chronic functional impairment, longitudinal assessments of HRQoL reveal a counterintuitive trajectory that perfectly encapsulates the “Toxicity-HRQoL Paradox” in melanoma care. Although the psychological relief of successful tumor control often overshadows physical toxicities—a phenomenon conceptualized as the ‘Toxicity-HRQoL Paradox’—group-level average scores can oversimplify individual patient outcomes. A clinically significant subset of survivors continue to experience severely compromised quality of life and long-term disability. In regional therapy cohorts, for instance, up to half of long-term survivors report persistent stiffness, edema, and numbness in the treated limb that cause permanent physical impairment [71,77]. Similarly, in systemic treatment cohorts, many survivors report stable overall global health scores while simultaneously suffering from chronic, treatment-limiting, or irreversible symptoms that prevent a complete return to baseline physical and social functioning [96,97].

Using the melanoma-specific FACT-M questionnaire, prospective studies demonstrate that while patients experience small, statistically significant decreases in Physical Well-Being and Melanoma Surgery Scale scores at 3 months post-procedure particularly those suffering from severe Wieberdink III-IV toxicity overall global HRQoL scores remain remarkably stable and generally rebound to baseline levels by 6 to 12 months [58,59,65].

This stability is largely attributed to the profound palliative benefits of treating in-transit metastases, which themselves cause significant morbidity, ulceration, bleeding, and pain if left unchecked [14,59,78]. By eradicating the symptomatic tumor burden, ILI and ILP provide immense psychological and physical relief. Paradoxically, studies note that despite the iatrogenic side effects of the surgery and regional infusion, fewer patients complain of pain, numbness, and swelling post-treatment compared to their baseline state when they were burdened by active, growing disease [65]. Ultimately, over the long term, the generic HRQoL (measured via the SF-36) of ILP survivors has been shown to be comparable to, or even significantly better than, that of the age- and gender-matched general population across domains such as bodily pain, mental health, and general health perceptions [77]. Table 3 aggregate quantitative data regarding locoregional treatments, specifically comparing SLNB, Complete/Inguinal Lymph Node Dissection (CLND/ILND), ILP/ILI. In this table we list the prevalence rates of resulting lymphedema, acute regional toxicities, and the corresponding point drops in physical functioning or FACT-M scores, demonstrating the high localized morbidity of these interventions.

### 3.2. Systemic Therapies: Historical Baselines vs. Modern Modalities

#### 3.2.1. High-Dose Interferon (HDI)

Before the advent of modern immunotherapy and targeted agents, high-dose interferon-alpha-2b (HDI) was the primary systemic adjuvant therapy for high-risk resected melanoma. However, its clinical utility was severely limited by frequent and sometimes irreversible toxicities [15,74,98]. In pivotal trials such as ECOG 1684, severe (Grade ≥ 3) adverse events occurred in 67–78% of patients, while up to 9% developed Grade 4 complications [74,98]. HDI toxicity primarily involved constitutional, neuropsychiatric, hepatic, and hematologic effects [99].

In ECOG 1684, Grade ≥ 3 constitutional symptoms occurred in 48.3% of patients, severe neurologic/neuropsychiatric events in 28.0%, myelosuppressive complications in 23.8%, and severe hepatotoxicity in 15.4% [98,100]. Depression affected 40–70% of patients, while suicidal ideation occurred in 5–10%. Early HDI-related hepatotoxic deaths led to stringent liver-function monitoring [74].

This substantial toxicity resulted in marked and prolonged deterioration of health-related quality of life (HRQoL), establishing the historical “Toxicity-HRQoL” relationship. EORTC QLQ-C30 assessments showed a pronounced reduction in global QoL during the initial 4-week intravenous induction phase [101]. During the subsequent 48-week maintenance phase, global QoL remained approximately 22% below baseline, with fatigue and depression representing major contributors to impaired physical and role functioning [101,102]. More broadly, HDI was associated with profound and prolonged negative effects on QoL, including chronic fatigue, severe pyrexia, hepatotoxicity, and depression, with some effects persisting beyond treatment completion [92,99,101].

Health-economic analyses further demonstrated the burden of HDI. In Q-TWiST models and standard gamble assessments, severe HDI toxicity was assigned a health utility of approximately 0.52 on a 0–1 scale, indicating patients’ willingness to trade survival time to avoid debilitating treatment-related effects [103]. Thus, the HDI era established a strong negative association between Grade ≥ 3 systemic toxicity and HRQoL and provides an important historical benchmark for evaluating contemporary adjuvant therapies.

Modern clinical practice has largely abandoned HDI in favor of more effective systemic immunotherapies and targeted therapies, which have substantially altered the relationship between treatment efficacy, toxicity, and HRQoL. In particular, adjuvant anti-PD-1 monotherapy with pembrolizumab or nivolumab generally preserves long-term HRQoL, with stable functioning and symptom scores comparable to placebo and approaching general population norms [52]. By contrast, although adjuvant CTLA-4 inhibition with ipilimumab improves survival, it is associated with a substantially greater symptomatic burden, including clinically meaningful acute deterioration in fatigue, diarrhea, and insomnia, particularly during the first 10 weeks of treatment [41,104].

#### 3.2.2. Immune Checkpoint Inhibitors (ICIs)

The introduction of ICIs specifically those targeting CTLA-4 (ipilimumab) and PD-1 (nivolumab and pembrolizumab) has radically transformed melanoma outcomes, but it has also introduced a completely novel toxicity profile. By removing inhibitory “brakes” on the immune system to unleash an anti-tumour response, ICIs frequently cause a loss of self-tolerance, resulting in collateral inflammatory damage to healthy tissues known as irAEs [19,81]. In the acute phase of treatment, the incidence of severe (Grade ≥ 3) irAEs is substantial, particularly with anti-CTLA-4 agents or combination therapies. For instance, in the pivotal EORTC 18071 trial evaluating adjuvant ipilimumab, 54% of patients experienced Grade 3 or 4 AEs predominantly gastrointestinal (e.g., severe colitis, 15.9%), hepatic (e.g., hepatitis, 10.6%), and endocrine which ultimately forced 52% of patients to discontinue treatment entirely [41,104]. While anti-PD-1 monotherapies demonstrate a more favourable acute safety profile, they still pose a considerable risk of severe systemic inflammation, including pneumonitis, nephritis, and enteritis, requiring rigorous monitoring and the prompt administration of systemic corticosteroids [39,48,81].

Beyond acute toxicities, extended survivorship has unmasked a growing prevalence of chronic and late-onset irAEs that cast a long shadow over the post-treatment continuum. Unlike the reversible side effects of traditional chemotherapy, certain ICI-induced toxicities inflict irreversible tissue damage. Chronic irAEs defined as those persisting at least 12 weeks after ICI cessation are estimated to occur in approximately 40% to 46.2% of patients [48,97]. The most profound of these are permanent endocrinopathies, such as hypophysitis (inflammation of the pituitary gland), thyroiditis, and primary adrenal insufficiency, as well as autoimmune diabetes; these conditions demand lifelong hormonal supplementation and ongoing medical management [43,48,96]. Furthermore, late-onset irAEs can emerge months or even years after treatment completion. Recent multicentre data revealed that nearly 15% of patients with advanced melanoma develop late-onset toxicities (including arthritis, late colitis, and neurological events) long after their final ICI infusion, underscoring a persistent, smouldering autoimmunity [47].

Despite this high burden of acute and chronic toxicity, the aggregation of HRQoL data across large clinical cohorts reveals a striking phenomenon: the preservation of global health status and functional capacity, perfectly illustrating the “Toxicity-HRQoL Paradox”. In the aforementioned EORTC 18071 trial, despite the severe toxicity that led to a 52% discontinuation rate for adjuvant ipilimumab, there were absolutely no clinically relevant differences (defined as a ≥10-point drop) in global health status scores between the ipilimumab and placebo arms after the induction phase [41]. While specific symptoms like diarrhoea and fatigue transiently spiked during active treatment, global HRQoL remained stable. Similarly, long-term analyses from the KEYNOTE-054 trial demonstrated that adjuvant pembrolizumab did not negatively affect long-term HRQoL or functional scale scores over 48 months of follow-up, even among the subset of patients who experienced irAEs [43,52].

This robust preservation of HRQoL is repeatedly validated in real-world metastatic cohorts as well. Studies tracking patients on highly toxic combined immunotherapy (ipilimumab plus nivolumab) show that, even when 51.4% of patients suffer from irAEs, their physical and mental component scores on standard instruments (like the SF-36 and EORTC QLQ-C30) remain remarkably constant throughout and after therapy [29,35]. The stability of these scores is heavily attributed to the psychological relief afforded by deep, durable tumour responses and extended recurrence-free intervals. Furthermore, researchers hypothesize that in the context of modern immunotherapy, patients may subconsciously perceive manageable immune-related side effects as reassuring physical evidence that the treatment is actively fighting the cancer, thereby psychologically offsetting the detriments of physical toxicity [41]. Consequently, while the physiological “price” of ICI therapy is undeniably high, its overall negative impact on the patient’s perceived quality of life is profoundly mitigated by the survival benefit it confers.

In real-world cohorts of melanoma survivors, chronic fatigue and joint pain (arthralgia) represent the most frequent and debilitating long-term toxicities, severely restricting physical, role, and social activities [29,97]. Because these symptoms are often chronic and difficult to manage, they impose a massive, long-term burden on healthcare systems. Patients suffering from persistent irAEs require lifelong endocrinology, rheumatology, or pain management specialist care, resulting in elevated clinic visit frequencies, repeated diagnostic imaging, and permanent reliance on expensive replacement medications or immunosuppression [47,105].

#### 3.2.3. Targeted Therapies (BRAF/MEK Inhibitors)

The introduction of small-molecule targeted therapies, specifically BRAF inhibitors (vemurafenib, dabrafenib, encorafenib) and their subsequent combination with MEK inhibitors (trametinib, cobimetinib, binimetinib), established a highly efficacious treatment paradigm for the approximately 50% of melanoma patients harboring a BRAF V600 mutation [1,13,85,106]. While the dual blockade of the MAPK pathway delayed the onset of biological resistance and improved survival outcomes, it also introduced a distinct, high-frequency toxicity profile [1,85]. Across landmark phase III trials, AEs of any grade were reported in over 90% of patients treated with combination TT, with severe (Grade ≥ 3) AEs occurring in 46% to 69% of patients, frequently necessitating dose interruptions or reductions [1,49,85]. The defining toxicities of this class include pyrexia (fever), severe dermatological reactions (such as rash, pruritus, and photosensitivity), arthralgia, and gastrointestinal disturbances like nausea and diarrhea [29,106,107].

Among these, pyrexia and dermatological toxicities are the most prominent drivers of treatment burden. Pyrexia is particularly prevalent with the dabrafenib plus trametinib combination, affecting up to 53% of patients and serving as the leading cause of dose modifications, though alternative combinations like encorafenib plus binimetinib report lower incidences (~18%) [1,107]. Skin toxicities uniquely demonstrate the mechanistic effects of targeted agents; while BRAF inhibitor monotherapy frequently induced secondary squamoproliferative lesions (like cutaneous squamous cell carcinomas and keratoacanthomas) via paradoxical MAPK activation in wild-type cells, the addition of a MEK inhibitor significantly reduced these hyperproliferative secondary malignancies [1,84]. Despite the reduction in secondary cancers, patients on combination therapy continue to report a high subjective burden of dry skin, acneiform rash, and muscle or joint aches [29,85].

Real-world registry data demonstrate that early, toxicity-related treatment discontinuation is a major determinant of poor long-term HRQoL. While patients who complete their scheduled adjuvant therapy show stable or rising quality of life trajectories, those who discontinue treatment early due to severe AEs demonstrate a progressive and significant decline in QoL over time [96]. This decline is heavily driven by the persistence of the severe toxicities that triggered the discontinuation, which continue to compromise physical and emotional functional domains long after drug clearance [35,96].

Despite this formidable landscape of acute physical toxicities, longitudinal patient-reported outcomes reveal a striking “Toxicity-HRQoL Paradox”: targeted therapies consistently demonstrate statistically significant improvements or remarkable stability in HRQoL and symptom burden when compared to historical benchmarks. In the BREAK-3 trial comparing dabrafenib against the historical standard dacarbazine chemotherapy, patients receiving dacarbazine experienced worsening in almost all functional and symptom dimensions, particularly fatigue and nausea [56]. Conversely, patients receiving dabrafenib showed rapid, clinically meaningful improvements in emotional and social functioning, appetite loss, and insomnia by week 6, proving that the high tumor response rates of BRAF inhibition quickly translate into tangible symptomatic relief [29,56].

Furthermore, the addition of MEK inhibitors not only enhances survival but also further improves QoL compared to BRAF monotherapy. The COMBI-v trial demonstrated that the combination of dabrafenib and trametinib yielded statistically significant and clinically meaningful improvements in global health status and multiple functional domains compared to vemurafenib alone [42,108]. This stabilization of HRQoL holds true even in the adjuvant setting (COMBI-AD), where asymptomatic patients undergo 12 months of therapy to prevent recurrence. Despite recurrent episodes of pyrexia, longitudinal EQ-5D-3L utility and VAS scores remained perfectly stable throughout the treatment phase [22,107]. Detailed analyses showed no clinically meaningful deterioration in QoL between those who experienced pyrexia and those who did not, as these acute events were typically transient (lasting a median of 3 days) and effectively managed with temporary dose interruptions [107]. Ultimately, the dramatic reduction in disease burden and the psychological relief of delayed progression effectively offset the high incidence of systemic toxicities, preserving the patients’ overall quality of life across both the metastatic and adjuvant continuums. Table 4 captures the “toxicity-HRQoL Paradox” by summarizing the mean changes in global HRQoL scores (primarily EORTC QLQ-C30 and EQ-5D) during adjuvant, neoadjuvant, and metastatic treatments with ICIs and targeted therapies. This data contrasts the high incidence of Grade ≥ 3 toxicities with the stable or even improved longitudinal HRQoL scores, illustrating how the psychological relief of survival and disease control often offsets the physical symptom burden.

Targeted therapies utilizing combined BRAF/MEK inhibitors (e.g., dabrafenib plus trametinib, vemurafenib plus cobimetinib, or encorafenib plus binimetinib) offer rapid disease control but introduce distinct, chronic, and long-term toxicities. Survivors face persistent cutaneous side effects, including hyperkeratosis, dry skin, severe photosensitivity, and an ongoing risk of secondary cutaneous squamous cell carcinomas or keratoacanthomas, which require vigilant dermatological monitoring [1,51]. Additionally, chronic pyrexia, persistent arthralgias, and neuromuscular complications (such as peripheral neuropathies and myopathies) severely limit daily functioning and require chronic dose adjustments [49,107]. Finally, long-term cardiotoxicity, characterized by chronic left ventricular ejection fraction (LVEF) reductions, hypertension, and arrhythmias, represents a delayed hazard that is frequently exacerbated by drug–drug interactions with patients’ concomitant medications [85].

### 3.3. The Long-Term Survivorship Burden

#### 3.3.1. Persistent and Late-Onset Adverse Events

As the therapeutic success of modern ICIs continues to translate into extended survivorship for patients with cutaneous melanoma, the clinical focus is increasingly expanding beyond acute toxicities to address the chronic sequelae of prolonged immune activation. While early-onset irAEs are well-characterized and often resolve with prompt corticosteroid administration or treatment interruption, a significant proportion of patients experience persistent, irreversible toxicities that mandate lifelong medical management [43,48]. Chronic irAEs typically defined as AES persisting for at least 12 weeks or 3 months following the discontinuation of ICI therapy are surprisingly prevalent. Retrospective analyses and European cohort studies estimate that between 35% and 46.2% of melanoma patients treated with ICIs will develop chronic irAEs [29,48].

The most profoundly life-altering among these chronic irAEs are endocrinopathies, such as hypophysitis, autoimmune thyroiditis, primary adrenal insufficiency, and type 1 diabetes mellitus. These conditions are characterized by the irreversible burnout or destruction of endocrine cells, rendering the patient permanently dependent on lifelong hormonal supplementation [43,48,50,97]. In addition to endocrine dysfunction, patients frequently suffer from persistent neuropathies, nephritis, and chronic dermatological conditions such as vitiligo and pruritus, all of which reflect sustained neuronal or tissue damage [29,48]. Even with extended follow-up spanning several years, nearly 30% of patients continue to present with persistent irAEs, many of whom remain symptomatic and require prolonged systemic immunosuppression, highlighting a substantial, ongoing burden on their HRQoL [48].

Further complicating the survivorship landscape is the phenomenon of delayed immune-related events (DIRE) toxicities that emerge *de novo* long after the patient has ceased active immunotherapy. Late-onset irAEs, defined as those appearing at least 3 months post-treatment, occur in approximately 15% of patients whose disease is otherwise under long-term control [47,48]. These delayed events manifest heterogeneously, frequently presenting as skin rash, colitis, hepatitis, or arthritis, and often necessitate the re-initiation of systemic steroids [47].

More remarkably, evidence is mounting regarding “ultra-late-onset” toxicities, which appear at least 12 months after the final ICI infusion. Recent multicenter data indicates that up to 2% of patients develop these ultra-late events [47]. The pathogenesis of these delayed toxicities is not entirely understood but is hypothesized to stem from a smoldering, subclinical baseline inflammation that eventually crosses a clinical threshold into autoimmunity [48]. These late and ultra-late toxicities represent a significant diagnostic hazard; because long-term survivors typically undergo less intensive clinical monitoring (often with follow-up appointments spaced 3 to 6 months apart), emerging symptoms may be overlooked, misattributed to other causes, or diagnosed at an advanced severity [47]. Consequently, the potential for permanent and late-emerging toxicities challenges the notion of a completely “disease-free” survivorship, underscoring the critical need for continued vigilance and tailored patient-reported outcome measures long after melanoma therapy has concluded [43,47].

#### 3.3.2. Psychosocial and Socioeconomic Impacts

Given the heavy psychosocial burden of melanoma, integrating formal distress screening and psychological support strategies into routine care is vital. Pretreatment emotional distress is highly prevalent, and in patients undergoing intensive cellular therapies (such as TILs), elevated baseline distress is strongly associated with a significantly lower probability of achieving an objective response (adjusted OR: 0.28) [38]. Thus, proactive psycho-oncological interventions are essential to maximize both clinical efficacy and mental well-being [28,35].

Furthermore, fear of cancer recurrence (FCR) remains a chronic stressor, particularly for women and younger survivors [67,110]. Survivorship programs should routinely implement validated FCR screening tools and nurse-led self-examination education to empower patients and reduce psychological worry [32,33]. Crucially, unresolved psychological distress and physical disability directly trigger work limitations, job loss, or early retirement, creating a socioeconomic loop where poor HRQoL actively exacerbates severe financial toxicity and limits resources for long-term supportive care [40,64].

A comprehensive meta-analysis of over 12,400 melanoma patients reveals that psychological distress fluctuates significantly across different survival and treatment milestones [28,38]. Prior to and during active treatment, the pooled prevalence of clinically significant anxiety reaches approximately 19%, before declining to 14–15% after treatment completion [28,38]. Similarly, depressive symptoms peak during active treatment at 12%, eventually dropping to 8% in the first year of survivorship for early-stage patients, though it remains persistently high (14%) for those with late-stage (Stage III and IV) disease [28]. Furthermore, female patients and younger individuals consistently report higher rates of intrusive thoughts, clinical anxiety, and distress compared to their male and older counterparts, indicating distinct demographic vulnerabilities in psychosocial adaptation [28,38,66,110]. Table 5 synthesizes data on which specific patient populations are most vulnerable to HRQoL deterioration presenting risk factors such as age, sex, socioeconomic status, and specific surgical interventions (e.g., lymph node involvement).

Beyond generalized anxiety and depression, Fear of Cancer Recurrence (FCR) emerges as the most pervasive and enduring psychosocial burden in melanoma survivorship [114]. Even following successful treatment and the achievement of durable responses with modern immunotherapies, a staggering 69% to 86% of survivors report moderate to severe fears of melanoma recurring or progressing [29,114]. This chronic uncertainty is frequently cyclical, becoming particularly pronounced in the weeks leading up to scheduled surveillance imaging and oncology appointments [29]. Elevated FCR is not merely an isolated psychological symptom; it is directly associated with maladaptive reassurance-seeking behaviors, increased physical fatigue, and significant decrements in emotional, role, and social functioning [55,114]. A comprehensive aggregation of the hidden burdens of surviving melanoma is presented in Table 6 compiling reported prevalence rates for clinically significant anxiety, depression, FCR, sleep disturbances, cognitive impairments, and financial toxicity. It also includes identified demographic risk factors (e.g., sex differences, younger age) for these psychological and socioeconomic impacts.

Compounding this psychological distress is the profound socioeconomic impact of surviving advanced melanoma, commonly referred to as “financial toxicity”. The high costs associated with modern cancer care, combined with ancillary expenses such as travel and accommodation for treatment at specialized academic centers, create a substantial economic strain on households [29,40,109]. Furthermore, treatment-related toxicities—most notably chronic fatigue and cognitive impairments—frequently force patients to reduce their working hours or prematurely exit the workforce entirely [109,115]. This loss of employment not only exacerbates financial pressure through lost wages but also triggers a profound loss of personal identity and self-esteem, severely compounding the patient’s overall psychosocial burden [29,109].

Quantitative evaluations of financial toxicity such as those utilizing the comprehensive score for financial toxicity (COST) instrument demonstrate a direct, inverse correlation between economic burden and HRQoL [40,64]. Studies indicate that financial toxicity is significantly higher among younger patients (<65 years of age) who are typically at the peak of their earning potential and face greater disruptions from employment loss and diminished retirement savings [29,64]. Crucially, independent of patient age, worsening financial toxicity strongly correlates with statistically significant deteriorations in almost all EORTC QLQ-C30 functioning subscales, including global health status, physical functioning, emotional functioning, and social functioning [64]. Thus, the economic burden of modern melanoma care is deeply intertwined with the patient’s subjective well-being, highlighting that achieving long-term survival must also be evaluated against the socioeconomic and psychological tolls exacted across the treatment continuum. Table 6 synthesizes the objective health-economic data alongside the subjective patient experience of financial toxicity. In this table we compare the incremental cost-effectiveness ratios (ICERs) and cost per QALY across different treatments, such as adjuvant pembrolizumab versus observation, HDI versus watchful waiting, and surgical interventions like sentinel node biopsy (SNB) versus wide excision (WE).

## 4. Discussion

### 4.1. Decoding the Toxicity-HRQoL Paradox

#### 4.1.1. The Trade-Off Between Survival and Toxicity

Although the psychological relief of successful tumor control often overshadows physical toxicities—a phenomenon conceptualized as the “toxicity-HRQoL paradox”—relying solely on group-level statistical averages risks oversimplifying individual experiences. A clinically significant subgroup of survivors continue to suffer from severely compromised quality of life and long-term disability despite achieving complete oncologic disease control. In surgical and regional therapy cohorts, patients often experience persistent, highly debilitating physical limitations (including severe lymphedema, limb stiffness, and neuropathic pain) that permanently restrict daily role and social functioning [70,77]. Similarly, in systemic therapy cohorts, stable overall global health scores frequently mask severe, ongoing physical or organ-specific toxicities that prevent a complete return to baseline well-being [97].

Patients diagnosed with advanced cutaneous melanoma are frequently confronted with a profound clinical dilemma: balancing the prospect of prolonged survival against the risk of severe, and sometimes permanent, treatment-related toxicities [26,39,116]. In the metastatic setting, the immediate threat of a historically fatal disease shifts patient preferences heavily toward maximizing therapeutic efficacy. Consequently, patients often demonstrate a remarkable willingness to accept a high degree of physical morbidity, including irreversible irAEs or debilitating TT toxicities, in exchange for improved survival chances and disease control [26,100,116]. For instance, studies suggest that a significant majority of oncology patients prefer a “hopeful gambling” approach selecting highly toxic treatments that offer a chance at long-term durable survival over “safe bet” palliative treatments that offer marginal survival benefits with fewer side effects [26].

This trade-off is deeply influenced by the clinical context and the patient’s individual valuation of health states. Historical health-economic models, such as the Q-TWiST, quantitatively established that patients are willing to trade substantial periods of their life experiencing severe toxicity to gain time free from disease relapse [100]. In the modern era of immunotherapy, this phenomenon persists strongly. Studies evaluating patient decision-making reveal that those who actively choose to undergo adjuvant systemic therapy to reduce the risk of recurrence report significantly less decisional regret compared to those who opt for observation, even if they subsequently experience severe irAEs or cancer recurrence [116]. However, the acceptance of these permanent risks (such as lifelong endocrinopathies requiring hormone replacement) is more complex in the adjuvant setting, where patients are macroscopically tumor-free after surgery and already have a substantial possibility of remaining disease-free without additional intervention [41,116].

The willingness to endure these toxicities underpins the “Toxicity-HRQoL Paradox”, wherein high incidences of AEs do not proportionately degrade global health status scores. This paradox is largely explained by the fact that the psychological relief of survival effectively counterbalances the physical symptom burden [29,36,41]. Before the advent of modern therapies, the diagnosis of metastatic melanoma was accompanied by severe existential distress, fear of imminent death, and a bleak prognosis [8,109]. When modern treatments induce rapid tumor regression or significantly delay recurrence, patients experience a dramatic reduction in this disease-related psychological burden. The intermittent periods of clinical certainty and reassurance gained from positive imaging scans and ongoing treatment response provide immense psychological comfort, which offsets the daily detriments of physical fatigue, gastrointestinal issues, or dermatological toxicities [29,56,109].

Furthermore, psychological adaptation and cognitive reframing play a crucial role in mitigating the impact of treatment toxicities on overall well-being. It has been hypothesized that patients may subconsciously perceive manageable immune-related or TT side effects as reassuring, tangible evidence that the systemic therapy is actively engaging the immune system or aggressively fighting the malignancy [35,41]. Over time, successful illness coping processes and psychological adaptations become widespread among long-term survivors, allowing them to integrate chronic symptoms into their daily lives without experiencing a catastrophic collapse in their perceived quality of life [36,55]. Ultimately, while the physical price of survival is high, the overwhelming value patients place on life extension and the alleviation of cancer-related existential distress predominantly drives the preservation of their global HRQoL [36,41,116]. Table 7 specifically aggregates “Utility Scores” (typically measured via EQ-5D or Time Trade-Off methods on a 0 to 1 scale, where 1 is perfect health). Within Table 8 we listed the utility values assigned to various health states, such as “disease-free clinically,” “loco-regional relapse,” “distant metastases,” and “treatment with severe toxicity”. Such information is crucial for understanding the quantitative health-economic impact of AEs and survival.

#### 4.1.2. Methodological Limitations of Current HRQoL Instruments

While the Toxicity-HRQoL paradox is heavily driven by the psychological relief of survival, it is also partially an artifact of how HRQoL is currently measured in oncology. The widely utilized generic cancer instruments, such as the EORTC QLQ-C30 and the EQ-5D, present significant methodological limitations Potential for long-term cardiovascular complications (e.g., heart failure, arrhythmias) due to drug–drug interactions when evaluating modern melanoma treatments [27,41,52,96]. Developed primarily during the era of cytotoxic chemotherapy, these tools effectively capture classic acute symptoms such as nausea, vomiting, and fatigue [35,41]. However, they lack the specificity required to detect the unique and often chronic irAEs induced by ICIs, or the specific toxicities associated with targeted therapies [35,108]. Most notably absent from these generic questionnaires are items designed to assess permanent endocrine dysfunctions (such as hypophysitis, autoimmune thyroiditis, and adrenal insufficiency) and specific dermatological toxicities (including rash, vitiligo, and severe pruritus) [41,52,96].

Consequently, the reported stability in global health status might reflect a measurement blind spot, where the true physical burden of modern therapies is underreported simply because the right questions are not being asked [35,108]. Recognizing this critical gap, there is a growing consensus that standard cancer-specific patient-reported outcome measures must be supplemented with disease- and treatment-specific modules to accurately capture the modern survivorship experience [35,108]. For instance, tools like the EORTC QLQ-MEL38 or the FACT-ICM (Functional Assessment of Cancer Therapy—Immune Checkpoint Modulator) are currently being developed and validated to better quantify the granular, day-to-day impact of these novel toxicities [35,108].

However, their large-scale international validation in real-world, heterogeneous cohorts is still ongoing, and they are subject to limitations, including increased administrative burden for patients and a high susceptibility to selective missing data in long-term follow-up [29,97]. Until these targeted instruments are universally integrated into clinical trials and real-world registries, the apparent preservation of HRQoL must be interpreted with caution. Furthermore, future trial designs must look beyond classic clinical questionnaires and systematically incorporate broader survivorship metrics. These include evaluating patients’ capacity to return to work, their objective physical activity levels, and the burden of long-term disability, all of which are major determinants of societal reintegration and are closely linked to the severity of financial toxicity [39,64].

A second major methodological confounding factor in interpreting longitudinal HRQoL data is the psychological phenomenon known as the “response shift” [22,23,70]. A response shift occurs when patients diagnosed with a severe, life-threatening condition undergo a profound internal psychological readjustment [22]. As they confront their mortality and undergo intensive treatment, patients dynamically adapt to their illness by re-evaluating their internal standards, values, and overall conceptualization of what constitutes a “good” quality of life [22]. Essentially, the patient’s frame of reference shifts: a health state or physical limitation that a patient might have considered unacceptable prior to their cancer diagnosis may be newly perceived as satisfactory or even excellent when the alternative is disease progression or death [22,70].

This self-readjustment process significantly complicates the interpretation of the Toxicity-HRQoL paradox. Because patients with cancer experiences interpret and respond to HRQoL questions fundamentally differently than they would have before their diagnosis or differently than a healthy control population direct comparisons to baseline or normative data may lack strict validity [70]. Even when facing permanent toxicities, the response shift mechanism enables patients to integrate chronic symptoms into their daily lives without registering a catastrophic drop in their self-reported well-being [22]. Therefore, the stable or improved HRQoL scores frequently observed in modern melanoma trials are not merely a direct reflection of physical health, but represent a complex interplay between treatment efficacy, the methodological limitations of generic questionnaires, and the remarkable psychological resilience of patients adapting to survive [22,52,70].

To address the methodological confounding of response shift in longitudinal research, future studies should implement specific investigative strategies. First, trials should adopt the ‘then-test’ design, which retrospectively asks patients to re-evaluate their baseline quality of life after experiencing treatment, thereby capturing the internal shift in their standards of measurement [77]. Second, researchers should utilize advanced statistical modeling, such as longitudinal structural equation modeling (SEM) or latent class growth analysis, to mathematically isolate and quantify response-shift effects over time. Finally, integrating mixed-methods approaches that combine quantitative questionnaires with structured qualitative patient interviews will allow clinicians to contextualize self-reported scores and better understand how survivors adapt to their chronic toxicities [29].

### 4.2. Neoadjuvant vs. Adjuvant Approaches

#### 4.2.1. Comparative HRQoL Outcomes

The traditional management of high-risk stage III cutaneous melanoma has historically relied on an adjuvant paradigm: upfront extensive surgery (often including therapeutic lymph node dissection [TLND]) followed by up to 12 months of systemic immunotherapy to eradicate micrometastases and prevent recurrence [16,18,25,30,44,45,46,48,83]. However, this approach exposes all patients including those who may have already been cured by surgery alone to a year-long risk of irAEs and leaves them in a state of psychological uncertainty regarding the actual efficacy of the treatment [18,24,25,44,46,48,83]. Recently, the treatment landscape has shifted toward neoadjuvant approaches, where ICIs are administered prior to surgery. This strategy not only induces high pathological response rates but also allows for a shorter duration of systemic therapy and the potential de-escalation of surgical interventions, thereby minimizing surgical morbidity [16,69,83,119,120]. Despite their exceptional pathologic response rates, a major limitation is that current evidence is derived from relatively small, early-phase trials with short follow-up periods. Consequently, robust long-term overall survival (OS) data comparing neoadjuvant versus adjuvant paradigms are still lacking, and larger, mature phase III randomized controlled trials (such as the NADINA trial) are urgently required to confirm long-term safety and survival benefits [69,83].

In addition, the pre-operative administration of systemic therapy induces a unique psychological duality in patients. On the positive side, achieving a pathologic complete response (pCR) or major pathologic response (MPR) provides immediate, profound psychological reassurance, significantly reducing fear of recurrence and empowering patients to accept de-escalated surgical plans [69,83]. On the negative side, the neoadjuvant window can trigger severe pre-operative anxiety and distress, as patients must cope with the acute physical toxicities of intensive dual immunotherapy while facing the persistent fear that their tumor may progress and prevent their planned curative surgery.

There is also emerging real-world data provide compelling evidence that this also shift translates into significantly superior long-term HRQoL outcomes. A recent cross-sectional analysis comparing stage III melanoma patients evaluated approximately 2.5 years after treatment initiation demonstrated that those who received neoadjuvant ICI reported clinically relevant and statistically superior HRQoL compared to their counterparts treated with standard adjuvant ICI [83]. Specifically, using the EORTC QLQ-C30 questionnaire, patients in the neoadjuvant cohort scored significantly higher in physical functioning (93 vs. 88), role functioning (93 vs. 84), cognitive functioning (86 vs. 77), and social functioning (92 vs. 85), while also reporting significantly less severe fatigue (13 vs. 23) [83]. Furthermore, multivariable regression analyses confirmed that receiving neoadjuvant rather than adjuvant ICI was an independent predictor of reduced long-term fatigue, effectively demonstrating a distinct survivorship advantage [83].

The mechanisms driving these superior functional outcomes in the neoadjuvant setting are multifactorial, encompassing both physical and psychological dimensions. Physically, the neoadjuvant approach facilitates surgical de-escalation; patients who achieve a major pathological response (MPR) can frequently safely omit TLND in favor of an index lymph node biopsy alone [83,119,120,121]. By avoiding extensive regional surgery, patients face a drastically reduced risk of developing chronic, debilitating lymphedema a complication known to severely restrict long-term physical mobility and role functioning [69,119]. Furthermore, neoadjuvant ICI regimens are completed much earlier (often within 6 to 9 weeks prior to surgery), facilitating a faster return to daily life, social activities, and the workforce compared to the protracted 12-month adjuvant treatment schedule [83].

Psychologically, the neoadjuvant setting provides a profound advantage that perfectly illustrates the “Toxicity-HRQoL Paradox”. While neoadjuvant combination immunotherapies (such as ipilimumab plus nivolumab) are associated with higher incidences of acute Grade 3 or 4 toxicities compared to adjuvant anti-PD-1 monotherapy, these acute physical detriments do not result in long-term HRQoL impairment [45,69,83]. This is largely because neoadjuvant therapy allows clinicians to clearly communicate the objective, pathological treatment response directly to the patient at the time of surgery [83]. The psychological relief and reassurance gained from achieving a MPR effectively counteract the distress associated with acute toxicities or persistent irAEs like endocrinopathies [48,83]. In contrast, patients receiving adjuvant therapy remain in a state of chronic uncertainty regarding whether the drug is actually working, leading to higher levels of psychosocial distress and poorer long-term functional scores [83]. Ultimately, these findings strongly advocate for the broader implementation of neoadjuvant ICI strategies to optimize both survival and long-term quality of life. Table 9 quantitatively compare the incidence of Grade ≥ 3 AEs, ORR, and Time to Recurrence (TTR) between neoadjuvant approaches (e.g., combined ipilimumab and nivolumab, targeted BRAF/MEK inhibition) and standard adjuvant therapies or surgery alone. This comparison shows whether administering therapy before surgery alters the toxicity profile or improves survival metrics compared to post-surgical administration.

#### 4.2.2. Surgical De-Escalation

The historical standard of care for macroscopic stage III cutaneous melanoma invariably mandated extensive surgical interventions, most notably TLND or CLND. While these procedures were intended to achieve locoregional disease control, they are inherently associated with profound and often irreversible morbidities, particularly chronic lymphedema, which significantly deteriorates long-term HRQoL by restricting limb mobility and causing persistent pain, body image issues, and psychosocial distress [70,71,75,87,88,95]. In recent years, the treatment paradigm has experienced a monumental shift with the introduction of highly effective neoadjuvant ICIs and targeted therapies. By administering these systemic treatments prior to surgical resection, clinicians aim to induce tumor shrinkage, eradicate micrometastases early, and most importantly, facilitate a tailored, de-escalated surgical approach that limits iatrogenic harm [69,83,121,123,125].

The potential for this surgical de-escalation is deeply rooted in the high pathological response rates observed following neoadjuvant therapy. Clinical trials have convincingly demonstrated that a substantial proportion of patients achieve a MPR or pCR after a brief course of neoadjuvant combination immunotherapy (e.g., ipilimumab plus nivolumab) or BRAF/MEK targeted therapies [21,24,119,124,126,127]. The landmark PRADO trial successfully established the clinical feasibility of personalized, response-directed surgery. In this study, patients who achieved an MPR in a marked index lymph node were able to safely omit the highly morbid TLND without compromising their relapse-free survival, effectively avoiding the complications of major surgery [75,118,119,127]. Furthermore, this strategy allows for the potential personalization of excision margins at the primary or regional site according to the degree of pathological response, converting formerly unresectable or highly morbid cases into candidates for conservative, tissue-sparing surgery [75,120,121,125,127].

The direct translation of surgical de-escalation into enhanced long-term HRQoL represents one of the most critical triumphs of the neoadjuvant approach. By safely omitting extensive regional lymphadenectomies, patients face a drastically reduced risk of developing debilitating lymphedema, thereby preserving the physical, role, and social functioning that are typically devastated by chronic limb swelling [69,75,83,119]. Recent cross-sectional analyses confirm that patients who undergo neoadjuvant therapies followed by tailored, less morbid surgery report clinically relevant improvements in physical and cognitive functioning, as well as significantly lower levels of long-term fatigue, compared to those who undergo upfront extensive surgery followed by a full year of standard adjuvant therapy [24,83,96,123]. Additionally, omitting complex surgeries expedites postoperative recovery, allowing patients to reintegrate into their daily lives and work much faster, thereby simultaneously alleviating the financial toxicity and psychological burden associated with prolonged, physically disabling treatments [48,64,83].

Despite the modern trend toward surgical de-escalation, extensive regional surgery remains critical for specific patient populations. Subgroups that still derive vital therapeutic and regional control benefits from radical inguinal or axillary lymphadenectomy include patients who present with clinically palpable, bulky macrometastatic disease, those who demonstrate non-response or progression on neoadjuvant therapy, and patients who are unable or unwilling to comply with the rigorous, high-frequency radiologic and ultrasound surveillance protocols required for nodal basin observation [75,128].

From an oncologic safety perspective, randomized trial evidence has validated the safety of omitting completion lymph node dissection (CLND) in patients with micrometastatic sentinel-node positive disease. Landmark trials (such as MSLT-II and DeCOG-SLT) demonstrated that omitting CLND does not compromise overall survival or disease-specific survival, provided that patients undergo rigorous, high-quality nodal basin surveillance (such as serial high-resolution ultrasound) to facilitate early, safe therapeutic dissection only if macroscopic recurrence occurs [75,118].

### 4.3. Multidisciplinary Management of Toxicities

#### 4.3.1. Proactive Dermatologic and Endocrine Care

The rapid expansion of targeted therapies and ICIs has significantly increased the complexity of cutaneous melanoma management, establishing the multidisciplinary team as the cornerstone of optimal patient care [8,105,128]. Because the skin is one of the most frequently affected organs during systemic therapy, dermatologic AEs such as severe maculopapular rashes, blistering, and therapy-induced secondary cutaneous malignancies constitute a major clinical challenge [1,51,84]. Historically, these toxicities have been predominantly managed by oncologists, with surveys revealing a notable hesitancy to request early dermatologic consultations unless symptoms become severe or persist for extended periods [1]. However, incorporating dermato-oncologists early in the treatment pathway is critical to correctly grading these complex toxicities, managing lesions in challenging anatomical sites (such as skin appendages), and providing comprehensive preventative counseling [1,105].

The proactive involvement of dermatologists is essential to prevent unnecessary dose reductions or the premature termination of life-saving systemic therapies. Severe dermatologic toxicities are a leading cause of treatment interruptions, which can inadvertently compromise therapeutic efficacy and allow for disease progression [1,84]. For instance, BRAF inhibitors can trigger the rapid development of squamoproliferative lesions, such as keratoacanthomas and cutaneous squamous cell carcinomas, while ICIs may exacerbate preexisting autoimmune dermatoses or cause profound inflammatory rashes [1,21,51,84]. Dedicated surveillance through joint dermato-oncology clinics enables the early identification and localized destruction of secondary malignancies, as well as the prompt initiation of targeted topical treatments for immune-related rashes [1,84]. Prospective evidence confirms that the timely and integrated management of cutaneous irAEs prevents their progression, thereby directly influencing the patient’s ability to safely remain on their oncology treatment regimen and maximizing their overall clinical benefit while preserving quality of life [51,128].

Beyond the acute phase, the multidisciplinary approach must be seamlessly extended into long-term survivorship to address persistent and late-onset toxicities, particularly those involving the endocrine system. Unlike many acute cutaneous or gastrointestinal toxicities that resolve with immunosuppression or treatment cessation, immune-mediated endocrinopathies such as hypophysitis, primary adrenal insufficiency, and autoimmune thyroiditis are typically characterized by the irreversible destruction of glandular tissue [48,125,128,129]. These fixed immune-related toxicities dictate a lifelong dependency on hormonal supplementation, mandating close, ongoing collaboration with endocrinologists to educate patients on the long-term effects of steroid use, manage complex comorbidities like therapy-induced diabetes, and regularly assess bone health [50,125,128,129].

Crucially, the conclusion of active immunotherapy does not signal the end of immune-related risks. The phenomenon of DIRE often presenting as late-onset or even ultra-late-onset toxicities months or years after the final infusion represents a significant diagnostic hazard, commonly described as “autoimmunity at a distance” [47,130]. Because these patients are typically seen less frequently during extended survivorship follow-up, late-emerging symptoms (whether they be delayed cutaneous rashes, pneumonitis, or arthritis) can easily be misattributed or overlooked until they reach advanced severity [31,32,33,34,47]. Consequently, continuous multidisciplinary monitoring is strictly necessary throughout the survivorship continuum. Clinical practice guidelines emphasize that the oncology team must perform regular, comprehensive reviews of systems and continually reinforce patient education, ensuring survivors remain vigilant in recognizing and rapidly reporting delayed AEs to safeguard their long-term health and quality of life [31,32,33,47,125,130].

#### 4.3.2. Survivorship, Toxicity Management, and Follow-Up Schedules in Multidisciplinary Context

To address these complex physical and psychological burdens, post-treatment care must transition toward integrated, multidisciplinary models. While the historical focus was on dermatological and oncologic surveillance, comprehensive survivorship teams must actively include clinical psychologists to routinely screen for and counsel patients suffering from FCR and anxiety, as well as specialized physiotherapists to proactively manage and rehabilitate patients with chronic lymphedema and joint dysfunction [28,72,105].

Within these clinics, toxicity management must be carefully balanced with patient safety. Systemic adjuvant therapies carry immediate risks of life-threatening toxicities, making proactive, algorithm-driven side-effect management—including rapid dose holds, dose reductions, and the timely administration of systemic corticosteroids or immunosuppression—mandatory to ensure patient safety without prematurely compromising therapeutic efficacy [39,107].

Finally, we recommend that follow-up care for low-risk survivors (such as AJCC stages IB–IIC) be restructured. Implementing stage-adjusted, reduced-frequency follow-up schedules led by specialized oncology nurses has been shown to be clinically safe, highly satisfactory for patients, and economically favorable, significantly reducing the burden on healthcare resources while empowering survivors through structured self-examination education [31,32,131].

### 4.4. Strengths and Limitations of the Study

A primary strength of this study is its comprehensive, quantitative synthesis of patient-reported outcomes (PROs), health-related quality of life (HRQoL) trajectories, and health-economic data across the entire cutaneous melanoma treatment continuum. Rather than focusing on a single treatment phase, this review uniquely contrasts historical surgical and regional morbidity against modern systemic treatment toxicities. Furthermore, by evaluating data from multiple validated generic and disease-specific instruments (including the EORTC QLQ-C30, EQ-5D, FACT-M, and COST), we successfully conceptualize the “toxicity-HRQoL paradox” to illustrate how survival-related psychological relief balances physical treatment burdens.

However, several limitations must be acknowledged.

1. Our review process excluded non-English publications, which may have omitted relevant regional data.
2. Our database search strategy primarily targeted clinical staging, treatment modalities, and general quality of life, which may have limited the immediate retrieval of articles focused on specific psychosocial or socioeconomic domains. Future investigations should expand this search framework to systematically include keywords related to anxiety, depression, survivorship, fear of recurrence, financial toxicity (e.g., the COST tool), and late-onset adverse events [28,64].
3. Much of the compiled literature on surgical, regional, and systemic treatments relies on retrospective cohort registries and observational studies, which are inherently prone to selection, referral, and reporting biases, as well as missing, incomplete, or inconsistently documented clinical data [11,47,132].
4. Standard generic HRQoL tools such as the EQ-5D-3L and EORTC QLQ-C30 suffer from inherent structural limitations, including ceiling effects and an inability to capture specific, modern immunotherapy-related toxicities (e.g., distinct endocrine dysfunctions, vitiligo, or severe pruritus).
5. Longitudinal HRQoL assessments are heavily confounded by the “response shift” phenomenon, wherein survivors undergo internal psychological adaptation to their life-threatening diagnosis, meaning stable self-reported scores may inadvertently mask severe physical or cumulative burdens.

Additionally, the exclusion of studies without full-text availability (such as conference abstracts or unpublished registry records) represents a source of potential publication bias, which may have omitted emerging or ongoing clinical trial data.

### 4.5. Future Directions

To advance the field and optimize long-term survivorship, future clinical research and practice must focus on several key areas. Standard oncology questionnaires must be supplemented with modern-therapy-sensitive, melanoma-specific modules. Clinical trials and real-world registries should systematically adopt newer validated tools, such as the EORTC QLQ-MEL38 and FACT-ICM, to eliminate measurement blind spots and capture the granular, day-to-day impact of immunotherapies.

Further prospective phase III trials are required to refine neoadjuvant systemic regimens and validate pCR or major pathologic response (MPR) as surrogate endpoints. This will safely facilitate personalized, response-directed surgical de-escalation (such as omitting therapeutic lymph node dissections), sparing patients from chronic, treatment-limiting morbidities like lymphedema without compromising oncologic safety.

Researchers must prioritize the prospective collection of toxicity data on delayed and ultra-late-onset immune-related adverse events (irAEs). Because these toxicities can manifest months or years after treatment discontinuation, survivors require continuous, long-term monitoring that extends far beyond the active treatment phase.

Comprehensive post-treatment care must transition toward integrated, multidisciplinary models. Survivorship clinics should actively coordinate oncologists, dermato-oncologists, endocrinologists, psychologists, and physiotherapists to proactively manage persistent and late-emerging toxicities. Additionally, routine FCR screenings and financial toxicity (such as the COST tool) must be standard practice to address the pervasive socioeconomic and psychosocial tolls of modern melanoma care.

Beyond standard approved regimens, next-generation trial designs must pivot toward highly personalized, translational immunotherapeutic strategies to optimize clinical benefit and overcome resistance. A premier frontier in this personalized landscape is the development of therapeutic mRNA-based vaccines, which are custom-designed for each patient based on their unique tumor mutational profile to target patient-specific neoantigens [48,122]. Promising data from early-phase trials (such as KEYNOTE-942 evaluating the individualized neoantigen therapy V940/mRNA-4157 combined with pembrolizumab) have demonstrated significant recurrence-free and distant metastasis-free survival benefits in resected, high-risk melanoma [122]. These therapeutic vaccines are delivered via lipid nanoparticles to prime host T cells against patient-specific mutational targets, presenting a synergistic mechanism when combined with standard immune checkpoint blockade [19,48]. Future prospective phase III trials (such as INTerpath-001) will clarify the long-term clinical and translational implications of these personalized vaccine-ICI combinations, potentially shifting the adjuvant paradigm toward highly precise, mutation-directed treatment plans that minimize off-target toxicity [48,125].

## 5. Conclusions

The therapeutic landscape for advanced cutaneous melanoma has undergone a monumental shift, transitioning the disease from an acutely fatal illness to a chronic, manageable condition characterized by extended survivorship. Historically, the “price of survival” was dictated by severe acute and localized morbidities, such as the chronic lymphedema resulting from extensive lymph node dissections, or the debilitating acute constitutional symptoms associated with high-dose interferon. Today, unprecedented survival gains achieved through ICIs and targeted therapies have fundamentally altered the survivorship experience. The treatment burden has transitioned away from acute surgical toxicity toward navigating a complex spectrum of chronic, late-onset, and often permanent AEs, such as lifelong endocrinopathies and persistent neuropathies. Despite these severe physical toxicities, patients frequently maintain a stable global HRQoL a phenomenon termed the “Toxicity-HRQoL Paradox” because the profound psychological relief of disease control effectively offsets the physical symptom burden. Crucially, real-world data demonstrate that post-treatment HRQoL is not uniform across survivor populations; instead, outcomes vary dynamically depending on disease stage, treatment type, toxicity severity, and patient age, with younger patients demonstrating a unique vulnerability to role and social functioning disruptions. Furthermore, early, toxicity-related treatment discontinuation represents a major clinical determinant of poor long-term well-being, as patients who stop systemic therapy prematurely frequently fail to return to physical baseline and experience a progressive, long-term decline in overall quality of life.

However, interpreting this apparent preservation of QoL requires acknowledging the methodological limitations of current assessment strategies. Widely utilized generic instruments, such as the EORTC QLQ-C30 and EQ-5D, were primarily developed during the era of cytotoxic chemotherapy and frequently fail to capture the specific chronic toxicities of modern immunotherapies, such as distinct endocrine dysfunctions or specific dermatological reactions. Consequently, there is a pressing need for the widespread clinical adoption of melanoma-specific and modern-therapy-sensitive HRQoL instruments, such as the FACT-M, EORTC QLQ-MEL38, or the FACT-ICM. Utilizing these targeted modules is essential to eliminate measurement blind spots and accurately quantify the granular, day-to-day impact of novel therapies on the modern survivorship experience. However, clinicians must remain cognizant of the inherent limitations of these newer tools, which include increased administrative and questionnaire-completion burden for patients, a susceptibility to selective missing data during long-term follow-up, and a current lack of robust, large-scale international validation in real-world heterogeneous cohorts.

As patient survival extends, the pervasive psychosocial and socioeconomic tolls exacted by melanoma care have become increasingly prominent. Survivors frequently grapple with severe FCR, cognitive impairments, and profound financial toxicity stemming from high healthcare costs and premature exits from the workforce. This underscores an urgent need for the integration of dedicated cancer survivorship programs and routine financial toxicity screenings into standard post-treatment care. Optimal survivorship management must move beyond simple disease surveillance to include proactive, multidisciplinary models of care that integrate dermato-oncologists and endocrinologists to continuously monitor and manage permanent and late-emerging toxicities. Survivorship care should incorporate evidence-based supportive care models, such as digitally supported skin self-examination platforms like ASICA to facilitate earlier detection of regional recurrences, personalized exercise interventions like i-Move to manage chronic fatigue, and routine psychological distress screenings (using tools such as the Distress Thermometer) to target FCR.

However, several systemic barriers to implementing such multidisciplinary care in routine clinical practice must be addressed. These include geographical and travel distance barriers for patients residing far from tertiary academic centers, the persistent socioeconomic burden of financial toxicity, and a severe shortage of specialized clinical resources—such as psycho-oncologists, lymphedema-specialist physiotherapists, and endocrinologists—in community oncology practices.

Moving forward, future investigations must prioritize the longitudinal tracking of delayed and ultra-late-onset irAEs, which can emerge years after ICI cessation and represent a significant diagnostic hazard. Additionally, clinical research should continue to optimize neoadjuvant treatment approaches to further facilitate personalized surgical de-escalation, minimizing iatrogenic harm such as lymphedema without compromising efficacy. Nevertheless, the potential limitations of neoadjuvant therapy must be acknowledged, including a current lack of long-term overall survival (OS) data from large-scale phase III trials, the clinical challenge of managing neoadjuvant non-responders, and the potential for heightened pre-operative anxiety and psychological distress as patients await surgery while undergoing intensive systemic treatment. Finally, prospective studies are needed to evaluate targeted psychosocial and socioeconomic interventions, ensuring that the remarkable survival extensions achieved in modern melanoma care translate into a genuinely high quality of life for all survivors.

## Figures and Tables

**Table 1:** Standardized instruments utilized for assessing HRQoL and psychosocial distress in melanoma.

Abbreviation/Code	Instrument	Subscales or Categories	Measured Variables	The “Gap” (Limitations in Melanoma Survivorship and QoL)	Refs.
Generic					
SF-36	Medical Outcomes Study Short Form 36	Physical functioning Vitality Social functioning General health Bodily pain Physical role Emotional role Mental health Physical and mental component scales	General health status Physical and mental functioning in everyday life	Broadly captures general health to allow comparisons with healthy populations Lacks sensitivity to specific symptoms induced by melanoma treatments Fails to capture the specific psychosocial burden of a skin cancer diagnosis	[62]
EQ-5D	EuroQol 5 Dimensions	Mobility Self-care Usual activities Pain/discomfort Anxiety/depression VAS	Overall health state Utility scores for health economics	Primarily a generic utility measure used for health economics Not sufficiently sensitive to the specific emotional distress of melanoma patients Fails to capture immunotherapy AEs	[22,41,42,43,52,62]
HADS	Hospital Anxiety and Depression Scale	Anxiety symptoms (7 items) Depressive symptoms (7 items)	Severity of anxiety Severity of depression	Focuses strictly on clinical psychological states Does not capture physical morbidity Misses melanoma-specific fears, such as fear of recurrence Does not capture social and functional well-being	[28,54,63]
COST	Comprehensive Score for Financial Toxicity	Financial distress/toxicity	Objective financial toxicity Subjective financial distress resulting from cancer treatment	Highly specific to financial and economic burdens Does not cover physical or emotional HRQoL Requires concomitant use with other tools to capture a holistic view of symptom burden	[29,64]
Cancer-specific					
EORTC QLQ-C30	European Organization for Research and Treatment of Cancer Quality of Life Questionnaire Core 30	5 functional scales (physical, role, emotional, cognitive, social) 3 symptom scales (fatigue, pain, nausea/vomiting) 6 single items (dyspnea, insomnia, appetite loss, constipation, diarrhea, financial impact) Global health/QoL scale	Cancer-specific symptom burden Functional well-being Global health status	Lacks specific modules for modern immunotherapies Frequently fails to capture irAEs (e.g., skin reactions, endocrine issues) Does not capture melanoma-specific psychosocial fears	[41,43,52,62]
Melanoma-specific					
FACT-M	Functional Assessment of Cancer Therapy—Melanoma	FACT-G (physical, social/family, emotional, and functional well-being) Melanoma Subscale (24 items) Melanoma Surgery Subscale	Melanoma-specific physical, emotional, and social well-being Surgery-specific concerns	Struggles to fully capture chronic psychological uncertainty regarding durable responses and long-term survivorship May not adequately measure the specific fear of recurrence triggered by modern systemic therapies	[22,29,58,62,65]
SCI/CARS	Skin Cancer Index/Concern About Recurrence Scale	SCI: cosmetic, emotional, and social function CARS: fear of recurrence across health, death, social role, and womanhood	Appearance perception and cosmetic concerns FCR Psychosocial and localized fears	Highly targeted on localized or specific psychosocial fears Neglects broader physical symptoms, such as systemic treatment toxicities Neglects general cancer survivorship issues, requiring concomitant use of generic questionnaires	[54,62,66,67]

Abbreviation: adverse events (AEs); concerns about recurrence scale (CARS); Functional Assessment of Cancer Therapy—General (FACT-G); fear of cancer recurrence (FCR); health-related quality of life (HRQoL); immune-related adverse events (irAEs); quality of life (QoL); skin cancer index (SCI); visual analogue scale (VAS).

**Table 2:** Comparative Incidence of Acute versus Chronic Adverse Events by Treatment Modality.

Treatment Modality	Acute/Early-Onset Toxicities	Chronic/Late-Onset Toxicities	Refs.
Surgery (SLNB, CLND)	• Seroma and hematoma • Wound infection and dehiscence/necrosis • Acute postoperative pain • Deep vein thrombosis	• Chronic lymphedema (significantly higher with CLND, affecting mobility and physical function) • Nerve injury resulting in sensory deficits or neuropathic pain • Chronic physical discomfort and functional limitations	[70,71,75]
ILP/ILI	• Marked acute limb toxicity (erythema, edema, blistering) • Muscle inflammation and ischemia • Compartment syndrome requiring fasciotomy • Peripheral neuropathy and pain	• Persistent joint stiffness (e.g., restricted motion of ankle or wrist) • Long-term limb numbness/paresthesia • Muscle atrophy and persistent limb edema • Chronic skin discoloration (dulling to a tanned/bronze appearance)	[76,77,78]
ECT	• Local side effects: transient edema, erythema, hyperpigmentation • Mild to moderate local pain • Superficial skin ulceration or scabs/necrosis (typically resolving in 1–2 months) • Low-grade systemic symptoms (e.g., fever, exhaustion)	• Generally very low chronic toxicity • Minor cosmetic issues (e.g., long-term post-inflammatory hyperpigmentation or scarring) • Rare instances of sensory or motor neuropathy	[61,79,80]
ICI	• Acute irAEs typically within the first 12 weeks: • Maculopapular rash and pruritus • Colitis and diarrhea • Hepatitis • Pneumonitis • Severe fatigue • Rare but severe acute events: myocarditis, meningitis/encephalitis	• Permanent/irreversible endocrinopathies (hypothyroidism, hypophysitis, adrenal insufficiency, type 1 diabetes mellitus) • Chronic arthritis and arthralgia • Vitiligo • Persistent neuropathy • Late-onset/ultra-late-onset irAEs appearing months or years after treatment discontinuation	[36,47,81,82,83]
TT (BRAF/MEK inhibitors)	• Pyrexia (highly prevalent with dabrafenib + trametinib) • Photosensitivity and rash • Secondary cutaneous malignancies (squamous cell carcinomas, keratoacanthomas; usually within the first 2–3 months) • Cardiovascular toxicities (QT-interval prolongation, decreased ejection fraction, hypertension) • Gastrointestinal symptoms (nausea, diarrhea)	• Overall lower rates of persistent AEs compared to ICIs • Long-term joint discomfort/arthralgia • Persistent dry and itchy skin • Potential for long-term cardiovascular complications (e.g., heart failure, arrhythmias) due to drug–drug interactions	[1,23,36,84,85]

Abbreviations: v-raf murine sarcoma viral oncogene homolog B (BRAF); completion lymph node dissection (CNLD); Electrochemotherapy (ECT); immune checkpoint inhibitors (ICI); iosolated limb perfusion (ILP); isolated limb infusion (ILI); immune-related adverse events (irAEs); mitogen-activated protein kinase (MEK); sentinel lymph node biopsy (SLNB); targeted therapy (TT).

**Table 3:** The impact of surgical and regional interventions on physical functioning and limb morbidity.

Locoregional Intervention	Prevalence of Lymphedema/Edema	Acute Regional Toxicities/Complications	Impact on Physical Functioning & QoL Scores
SLNB alone	• Generally low incidence; slight lymphedema reported in 6% of groin SLNBs and 12% of axillary SLNBs [91].	• Low overall postoperative complication rate (approx. 4% to 7.4%) [91]. • Seroma occurs in approximately 9.3% of cases [71].	• QoL and physical functioning remain stable and comparable to or better than normative populations [91]. • No significant long-term delay in returning to baseline physical condition scores [92].
CLND/ILND	• Significantly high; slight lymphedema in 64% of groin CLNDs vs. 8% of axillary CLNDs [91]. • Limb volume increase ≥ 5% observed in 72% of ilio-inguinal patients [93]. • Patient-reported limb edema reaches 55.9% at 6 months post-CLND [71].	• High overall acute complication rates ranging from 23% to 51.4% [71,92]. • Postoperative seroma occurs in 27% of CLND patients [71]. • Grade 2–4 long-term surgical AEs in 62% of patients [93].	• Axillary CLND is associated with an ~8-point drop in EORTC physical functioning and a ~12-point drop in role functioning compared to SLNB [91]. • Initial steep drop in QoL and Physical Condition scores at 3 months, though scores generally return to baseline over 12–24 months [92,93]. • Resulting chronic lymphedema causes significant, sustained point drops in overall global health, physical function, and body image scores [70,71].
ILP/ILI	• Acute post-procedure edema reported in 82% to 88% of patients [76]. • Long-term functional limb complaints (edema, stiffness) persist in 49% to 55% of survivors [77].	• High acute toxicity; Grade III regional toxicity (erythema, blistering) in 20% to 24% of patients; Grade IV toxicity in 4% [58,77]. • Acute compartment syndrome requiring fasciotomy in ~9% of ILI cases [76]. • Postoperative numbness (59%) and pain (59%) are common [76].	• At 3 months post-ILP, significant point drops observed in FACT-M Physical Well-Being (−2.3 points) and Melanoma Surgery Scale (−3.2 points); patients with severe (Wieberdink III-IV) toxicity had a −4.3 point drop in Functional Well-Being [58]. • Despite high acute morbidity, long-term functional impairment is minimal and FACT-M scores tend to rebound to baseline levels, especially if a complete disease response is achieved [58,76].

Abbreviations: adverse events (AEs); completion lymph node dissection (CLND); European Organisation for Research and Treatment of Cancer (EORTC); Functional Assessment of Cancer Therapy—Melanoma (FACT-M); inguinal lymph node dissection (ILND); isolated limb perfusion (ILP); isolated limb infusion (ILI); quality of life (QoL); sentinel lymph node biopsy (SLNB).

**Table 4:** Global health status and HRQoL trajectories in modern systemic therapies.

Treatment Setting & Regimen	Incidence of Severe (Grade ≥ 3) Toxicity	Longitudinal HRQoL & Global Health Status Trajectory	The “Toxicity-HRQoL Paradox” (Why QoL is Maintained)	Refs.
Adjuvant ICI: Ipilimumab (10 mg/kg)	54.1% of patients experienced Grade 3–4 irAEs, leading to treatment discontinuation in 52% of patients	Global Health Status scores remained similar to placebo. No clinically relevant deterioration (≥10 points) was observed during or after the induction phase, despite acute toxicity drops (e.g., diarrhea at week 10)	The prolonged RFS benefit compensates for the transient, severe toxicities. Once the acute toxicity phase resolves, patients’ QoL rebounds to baseline	[41,52]
Adjuvant Anti-PD1: Pembrolizumab/Nivolumab	Approximately 14.7% experienced Grade ≥ 3 treatment-related AEs with Pembrolizumab	GHQ scores remained stable over 2 years (mean change −2.2 points) and up to 48 months (mean change −0.56 points), with no clinically relevant differences compared to placebo	Even though treatment causes systemic side effects, it does not meaningfully impair overall HRQoL. The prevention of disease recurrence prevents the steep QoL drops associated with distant metastases	[43,52,96]
Adjuvant TT: Dabrafenib + Trametinib	Significant increase in toxicity compared to placebo (e.g., high incidence of pyrexia)	Treatment had no substantial negative effect on patients’ perception of health status measured by EQ-5D-3L VAS and utility scores during the 12-month treatment or in long-term follow-up	Disease recurrence causes a severe deterioration in EQ-5D-3L VAS scores. The dramatically reduced risk of relapse on combination therapy preserves long-term health status, offsetting the burden of treatment pyrexia and arthralgia	[107]
Neoadjuvant ICI: Anti-PD1 ± Anti-CTLA4	Associated with high Grade 3/4 toxicity rates but induces exceptionally high pathological response rates	Two years post-treatment, neoadjuvant patients reported clinically relevant and superior physical, role, cognitive, and social functioning, and significantly less fatigue compared to standard adjuvant ICI patients	A shorter, 6-week intense toxicity burst before surgery allows for a much faster return to normal life and better long-term HRQoL recovery than enduring 12 months of sustained adjuvant therapy	[83]
Metastatic ICI: Ipilimumab + Nivolumab	Grade 3–4 treatment-related AEs reported in 55% to 59% of patients	Despite intense toxicity, there were no clinically or statistically significant deteriorations in EORTC QLQ-C30 Global Health Status or EQ-5D-3L during treatment compared to baseline	The added adverse event impact of dual-ICI does not change the QoL compared to monotherapy. Unprecedented survival benefits and tumor shrinkage provide immense psychological relief that offsets the high physical toxicity	[26,29,109]
Metastatic TT: Dabrafenib + Trametinib	Frequent AEs including pyrexia (fever) and arthralgia, though combination therapy prevents secondary cutaneous malignancies seen in monotherapy	EORTC QLQ-C30 Global Health Status showed statistically significant and clinically meaningful improvements (+2.88 to +3.71 points) from baseline. Significant improvements were also seen in pain, insomnia, and appetite loss	The high tumor response rates and rapid disease control directly translate into rapid functional and symptomatic benefits. The alleviation of tumor-induced symptoms far outweighs the drug-induced toxicity profile.	[26,42,56]

Abbreviations: adverse events (AEs); cytotoxic t-lymphocyte-associated protein 4 (CTLA-4); European Organisation for Research and Treatment of Cancer (EORTC); EORTC quality of life questionnaire core 30 (EORTC QLQ-C30); EuroQol 5-dimension 3-level questionnaire (EQ-5D-3L); global health status (GHS); global health status/quality of life (GHQ); health-related quality of life (HRQoL); immune checkpoint inhibitors (ICI); immune-related adverse events (irAEs); programmed cell death protein 1 (PD-1); quality of life (QoL); recurrence-free survival (RFS); targeted therapy (TT); visual analogue scale (VAS).

**Table 5:** Demographic and clinical predictors of poor HRQoL and psychosocial distress.

Predictor/Risk Factor	Affected Patient Populations & Vulnerabilities	Quantitative Differences & Impact on HRQoL	Refs.
Age	**Younger patients (<65 years)** are significantly more vulnerable to anxiety, emotional distress, and financial toxicity, but have better physical functioning. **Older patients (>65 years)** report worse physical and role functioning but display better emotional/social functioning and a more positive body image.	Younger patients report higher financial toxicity and more financial difficulties compared to older patients (*p* < 0.001). Younger patients have worse HRQoL at baseline and report greater fear of recurrence, while older patients experience significantly less worry about the future. Older age at primary treatment is significantly associated with reduced anxiety and increased emotional well-being.	[63,64,67,110,111]
Sex	**Females** are at a much higher risk for clinical anxiety, distress, FCR, and body image dissatisfaction. **Males** tend to have better physical/emotional component scores but are more likely to have a pessimistic outlook regarding life expectancy.	27% of women report clinically significant high stress compared with 10% of men. Women report greater fear of future diagnostic tests, a second melanoma, recurrence, and metastasis compared to men. Men are more likely to agree with pessimistic statements that life’s duration is limited and time is running out (*p* < 0.0001).	[28,35,63,66,67,110]
Socioeconomic status and social support	**Lower educational attainment** and **poor social support** are strong predictors of psychological distress, depression, and high unmet supportive care needs.	Leaving school early (at 14–15 years of age) increases the odds of having unmet supportive care needs by nearly 5 times (OR = 4.85, *p* < 0.001). Lack of social support is linked to higher anxiety, higher depression, and significantly lower physical, functional, and emotional well-being.	[28,63,112]
Clinical factors: lymph node involvement and lymphedema	Patients with **regional lymph node involvement** and those who develop **limb lymphedema** after surgery suffer from worse global HRQoL, worse body image, and higher supportive care needs.	Patients with lymph node involvement report significantly higher unmet needs for physical, daily living, psychological, and sexual domains compared to those without nodal disease (*p* < 0.001). Melanoma-related limb lymphedema causes a clinically meaningful drop (≥5 points) in global health status, role functioning, social functioning, and body image. There is an interaction with age and sex: younger patients and women who develop lymphedema report significantly worse social functioning and poorer body image.	[70,71,112]
Clinical factors: comorbidities	A **higher number of physical comorbidities** is one of the strongest independent predictors of poor overall quality of life and depressive symptoms.	Regardless of tumor type, QoL significantly worsens with an increasing number of physical comorbidities. The presence of comorbid diseases explains the greatest unique variance in QoL outcomes, strongly correlating with worse vision-specific QoL, more depressive symptoms, and more concern about cancer recurring.	[63,111,113]

Abbreviations: fear of cancer recurrence (FCR); health-related quality of life (HRQoL); odds ratio (OR); quality of life (QoL).

**Table 6:** Prevalence of psychosocial, emotional, and socioeconomic burdens in melanoma survivorship.

Burden/Symptom Category	Reported Prevalence Rates	Identified Demographic & Clinical Risk Factors	Refs.
Anxiety	7% to 30% pooled prevalence depending on assessment timepoint and melanoma subtype. Highest prevalence (19%) observed before and during treatment. 29% reported signs of clinical or subclinical anxiety in a UK cross-sectional cohort. Distress Thermometer values ≥ 5 (indicating need for psycho-oncological support) seen in ~36% of long-term stage IV survivors.	Female sex: Women consistently report higher anxiety than men. Younger age: Associated with higher anxiety scores and greater unmet psychological needs. umor type & stage: Uveal melanoma (before treatment) and late-stage (III/IV) cutaneous melanoma correlate with higher anxiety.	[28,36,63,112]
Depression	6% to 16% pooled prevalence across various timepoints. 11% of patients reported clinical or subclinical depression in a UK stage I-III cohort. Severe depressive symptomatology reported in ~10% of women with localized melanoma.	Age differences: Older age is sometimes associated with increased depression (unlike anxiety), though younger patients also show high distress. Disease & comorbidities: Advanced stage (III/IV) and a higher number of physical comorbidities are strong predictors. Socioeconomic: Leaving school early and lacking social support increase risk.	[28,63,66,112]
FCR	81% to 86% of survivors report fear of melanoma recurring in observational studies of modern therapies. 29.2% report specific unmet needs regarding fears about the cancer spreading. Severe intrusive thoughts reported by 12% of localized melanoma patients.	Female sex: Women are significantly more fearful of recurrence, future diagnostic tests, and family members developing melanoma. Age: Younger patients (<65) experience greater distress and worse HRQoL regarding recurrence fears.	[29,66,110,112]
Fatigue and sleep disturbances	Fatigue: 35% pooled prevalence of clinically significant fatigue; ranges up to 90–92% in patients receiving targeted or immunotherapies. Insomnia/sleep disturbances reported by 35.2% to 62% of patients depending on the treatment cohort.	Age: Younger survivors (<40 years) show higher symptom burdens for fatigue compared to matched controls. Treatment: Strongly correlated with active or past receipt of systemic ICIs or targeted therapies.	[28,29,36]
Cognitive impairments	20% to 44% self-report cognitive complaints. Up to 56% report memory or concentration problems on the FACT-M questionnaire. 32.2% report memory/concentration issues on the NCCN problem list.	Treatment: Associated with prolonged systemic therapies (e.g., historical IL-2, modern ICIs).	[28,29,36]
Financial toxicity and socioeconomic burden	49% to 53% report difficulties managing financial affairs during/after systemic treatments. Financial toxicity (measured by COST) directly correlates with worse global quality of life. Only ~8% reported severe financial limitations in specific European cohorts (likely buffered by local social protection mechanisms).	Younger age: Patients < 65 years report significantly higher financial toxicity and distress about retirement/savings than older patients. Socioeconomic status: Lower baseline income and lower educational attainment heavily predict unmet socioeconomic needs.	[29,36,64,112]

Abbreviations: common terminology criteria for adverse events financial toxicity score (COST); ear of cancer recurrence (FCR); Functional Assessment of Cancer Therapy—Melanoma (FACT-M); health-related quality of life (HRQoL); immune checkpoint inhibitors (ICIs); interleukin-2 (IL-2); National Comprehensive Cancer Network (NCCN).

**Table 7:** Economic burden, cost-utility, and financial toxicity in melanoma care.

Treatment Comparison/Setting	Objective Health-Economic Data (ICER and Cost per QALY)	Subjective Patient Experience & Financial Toxicity Prevalence	Refs.
Adjuvant anti-PD1 (Pembrolizumab/Nivolumab) vs. observation	Highly cost-effective: For resected stage IIB/IIC melanoma, pembrolizumab yields an ICER of CHF 27,424/QALY (EUR 27,342/QALY), well below the WTP threshold of CHF 100,000. In Europe for stage III, ICERs were reported as €33,878 for pembrolizumab and €21,153 for nivolumab per QALY gained.	Significant financial distress: Among advanced melanoma patients treated with immunotherapy, 23% report financial difficulties. The mean COST score is 30.0, and financial toxicity is inversely associated with all QoL domains. Younger patients (<65 years) report significantly higher financial toxicity than older patients.	[48,60,64]
Adjuvant HDI vs. observation	Dependent on time horizon: In a Canadian model, the incremental cost per QALY for HDI was CAN$55,090 over a 7-year period but improved to an acceptable CAN$14,003/QALY when extrapolated over 35 years. US studies reported ICERs ranging from $US13,000 to $40,000 per life-year gained (1998 values).	Toxicity-driven burden: The high acute toxicity of HDI contributed heavily to medical resource use and hospitalizations. While subjective financial toxicity wasn’t strictly measured by COST, the severe reduction in QoL during the first year of treatment was an accepted trade-off for long-term survival gains.	[73,103]
SNB vs. WE alone	Not cost-effective: For intermediate thickness melanoma, SNB had an ICER of €130,508/QALY over 10 years, far exceeding the €30,000/QALY WTP threshold. For thick and thin melanomas, SNB was dominated by WE (meaning SNB was more expensive and less effective in terms of QALYs and life-years saved).	N/A (Focus in the literature is predominantly on objective hospital and procedural costs rather than subjective patient financial toxicity for these interventions).	[117]
Inguinal lymphadenectomy (IL) vs. ilio-inguinal lymphadenectomy (I-IL)	IL is the dominant strategy: For stage III patients with no pelvic disease on imaging, the less extensive IL surgery is slightly more effective and significantly less costly, saving the health system an average of AU$6938 per patient compared to the more extensive I-IL surgery.	N/A	[118]

Abbreviations: cost of illness therapy (COST); high-dose interferon (HDI); incremental cost-effectiveness ratio (ICER); inguinal lymphadenectomy (IL); ilio-inguinal lymphadenectomy (I-IL); programmed cell death protein 1 (PD-1); quality-adjusted life year (QALY); quality of life (QoL); sentinel node biopsy (SNB); willingness to pay (WTP); wide excision (WE).

**Table 8:** Patient health utility scores across melanoma health states.

Health State/Clinical Scenario	Elicitation Method and Instrument	Mean Utility Score (or Range)	Refs.
DFS/RFS	EQ-5D-5L Standard Gamble	0.9414 (EQ-5D-5L baseline) >0.90 (Standard Gamble)	[60,103]
Loco-regional relapse	EQ-5D-5L	0.9261	[60]
Distant metastases/advanced recurrence	TTO EQ-5D-5L	0.23 (TTO Mean; showing severe FCR, with a median of 0.08) 0.8868 (EQ-5D-5L pre- and post-progression)	[60,73,103]
Active systemic treatment (metastatic setting)	EQ-5D	0.830 (During 1st-line treatment) 0.801 (During subsequent lines of treatment)	[22]
Treatment with severe toxicity (e.g., high-dose IFN)	TTO	0.52 (Reflecting the high physical and emotional burden of historical adjuvant toxicity)	[73,103]
Post-surgical states (sentinel node biopsy & lymphedema)	EQ-5D	0.7475 (Overall post-SNB average) 0.7431 (With slight limb swelling) 0.7043 (With severe limb swelling/lymphedema)	[90]
Long-term post-lymphadenectomy (36 months post-surgery)	EQ-5D-5L	0.91 (Following Inguinal Lymphadenectomy) 0.88 (Following Ilio-inguinal Lymphadenectomy)	[118]
Superficial skin metastases (treated with ECT)	EQ-5D	0.773 (Non-ulcerated tumours) 0.655 (Ulcerated tumours)	[61]

Abbreviation: disease-free survival (DFS); electrochemotherapy (ECT); EuroQol 5-dimension questionnaire (EQ-5D); EuroQol 5-dimension 5-level questionnaire (EQ-5D-5L); fear of cancer recurrence (FCR); interferon (IFN); recurrence-free survival (RFS); sentinel node biopsy (SNB); time trade-off (TTO).

**Table 9:** Neoadjuvant vs. adjuvant therapy: comparative toxicities and clinical outcomes.

Treatment Comparison/Regimen	Incidence of Grade ≥ 3 AEs	Response Rates (ORR & Pathological Response)	Clinical Outcomes (TTR, EFS, or RFS)	Refs.
Neoadjuvant vs. adjuvant Pembrolizumab (SWOG S1801 Trial)	Not explicitly contrasted in terms of severe toxicity differences in current data, but generally well-tolerated.	N/A (Trial focused primarily on EFS endpoints rather than pathological response).	Superior Survival: Neoadjuvant (3 cycles) followed by adjuvant pembrolizumab resulted in a significantly higher 2-year EFS (72%) compared to adjuvant-only administration (49%) (HR = 0.58).	[75,83,122]
Neoadjuvant Ipilimumab + Nivolumab vs. adjuvant Nivolumab (NADINA Trial)	Higher toxicity: Grade ≥ 3 immune-related AEs occurred in 29.7% of the neoadjuvant combination group versus 14.7% in the adjuvant monotherapy group. Persistent irAEs (e.g., endocrinopathies) were 25.0% vs. 7.5%, respectively.	High response: Major Pathologic Response (MPR, ≤10% residual viable tumor) achieved in 59% of patients in the neoadjuvant arm.	Superior survival: 12-month EFS was 83.7% for the neoadjuvant arm vs. 57.2% for adjuvant-only. At 18 months, EFS was 80.8% vs. 53.9% (HR = 0.32).	[75,83,122]
Neoadjuvant vs. adjuvant Ipilimumab + Nivolumab (standard dose) (OpACIN Trial)	Equally high toxicity: Both neoadjuvant and adjuvant arms using the standard dose (Ipi 3 mg/kg + Nivo 1 mg/kg) experienced extremely high rates of Grade 3 AEs (90%).	Strong predictor: 78% pathologic response in the neoadjuvant arm. Notably, 0% of patients who achieved a pathologic response relapsed.	Potential benefit: At a median follow-up of 25.6 months, 4 out of 10 patients in the adjuvant arm relapsed, compared to only 2 out of 10 in the neoadjuvant arm.	[26,69,123]
Neoadjuvant Ipilimumab + Nivolumab (optimized flipped dosing) (OpACIN-neo & PRADO Trials)	Reduced toxicity: Adjusting to a “flipped dose” (Ipi 1 mg/kg + Nivo 3 mg/kg) drastically reduced Grade ≥ 3 irAEs from 40% to 20% while maintaining efficacy.	High response: Radiographic ORR of 60%. pCR of 57%, and total pathologic response of 77% to 80%.	Stable survival & de-escalation: Achieved 2-year EFS of 77–80%. Patients achieving MPR safely omitted TLND without increasing recurrence.	[69,75,83,123]
Neoadjuvant TT (BRAF/MEK inhibitors) vs. surgery alone (Amaria 2018a/NeoCombi) [124]	Moderate toxicity: Approximately 15% developed Grade 3 AEs, with the majority being manageable Grade 1–2 events.	Excellent early response: Achieved an ORR of 85% and a pCR of 49% to 58%.	Massive survival improvement: The trial was stopped early due to an “outrageously superior” RFS benefit: 19.7 months for neoadjuvant therapy versus 2.9 months for surgery alone (HR 0.016 to 0.02).	[26,69,123]

Abbreviation: adverse events (AEs); event-free survival (EFS); hazard ratio (HR); immune-related adverse events (irAEs); major pathologic response (MPR); overall response rate (ORR); pathologic complete response (pCR); recurrence-free survival (RFS); time to recurrence (TTR); therapeutic lymph node dissection (TLND).

## Data Availability

The original contributions presented in this study are included in the article. Further inquiries can be directed to the corresponding author.

## Abbreviations

The following abbreviations are used in this manuscript:

AEs	Adverse events
CARS	Concern about recurrence scale
CLND	Complete lymph node dissection
COST	Comprehensive score for financial toxicity
CTLA-4	Cytotoxic t-lymphocyte-associated protein 4
DFS	disease-free survival
DIRE	Delayed immune-related events
ECOG	Eastern cooperative Oncology Group
ECT	Electrochemotherapy
EFS	Event-free survival
EORTC	European Organization for Research and Treatment of Cancer
EORTC QLQ-C30	European Organization for Research and Treatment of Cancer Quality of Life Questionnaire Core 30
EQ-5D	Euroqol 5 Dimensions
EQ-5D-3L	EuroQol 5-dimension 3-level questionnaire
EQ-5D-5L	EuroQol 5-dimension 5-level questionnaire
FACT-G	Functional Assessment of Cancer Therapy-General
FACT-ICM	Functional Assessment of Cancer Therapy-Immune Checkpoint Modulator
FACT-M	Functional Assessment of Cancer Therapy-Melanoma
FCR	Fear of Cancer Recurrence
GHS	Global health status
GHQ	Global health status/quality of life
HADS	Hospital Anxiety and Depression Scale
HDI	High-Dose Interferon (or High-dose interferon-alpha-2b)
HRQoL	Health-Related Quality of Life
ICER	Incremental cost-effectiveness ratio
ICI	Immune checkpoint inhibitor
IL	Inguinal lymphadenectomy
I-IL	Ilio-inguinal lymphadenectomy
IL-2	Interleukin-2
ILI	Isolated limb infusion
ILND	Inguinal lymph node dissection
ILP	Isolated limb perfusion
irAEs	Immune-related adverse events
MEK	Mitogen-activated protein kinase kinase
MPR	Major pathological response
NCCN	National Comprehensive Cancer Network
OR	Odds ratio
ORR	Overall response rate
pCR	Pathological complete response
PD-1	Programmed cell death protein 1
QALY	Quality-adjusted life year
Q-TWiST	Quality-Adjusted Time Without Symptoms and Toxicity
RFS	Recurrence-free survival
SCI	Skin cancer index
SF-36	Medical outcomes study short form 36
SLNB	Sentinel lymph node biopsy
SNB	Sentinel node biopsy
TLND	Therapeutic lymph node dissection
TT	Targeted therapy
TTO	Time trade-off
TTR	Time to recurrence
VAS	Visual analogue scale
WE	Wide excision
WTP	Willingness-to-pay
